# Supplementary figures and images for: SRSF2 is a key player in orchestrating the directional migration and differentiation of MyoD progenitors during skeletal muscle development
Source: eLife. 2024 Sep 9;13:RP98175. doi: 10.7554/eLife.98175 (PMC11383525; doi:10.7554/eLife.98175)

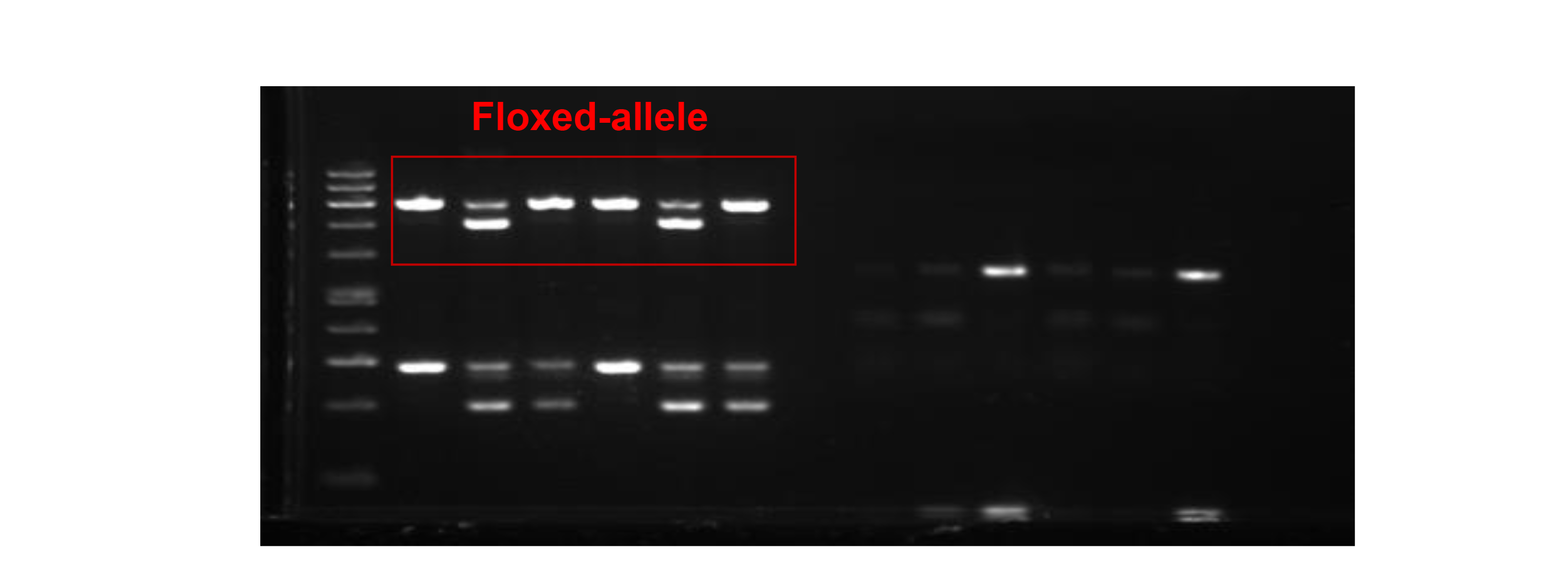

Supplement: Figure 1—figure supplement 1—source data 1. [file elife-98175-fig1-figsupp1-data1.zip › Fig 1-supplement 1B source data 1/Figure1-supplement 1B floxed allele.tif]

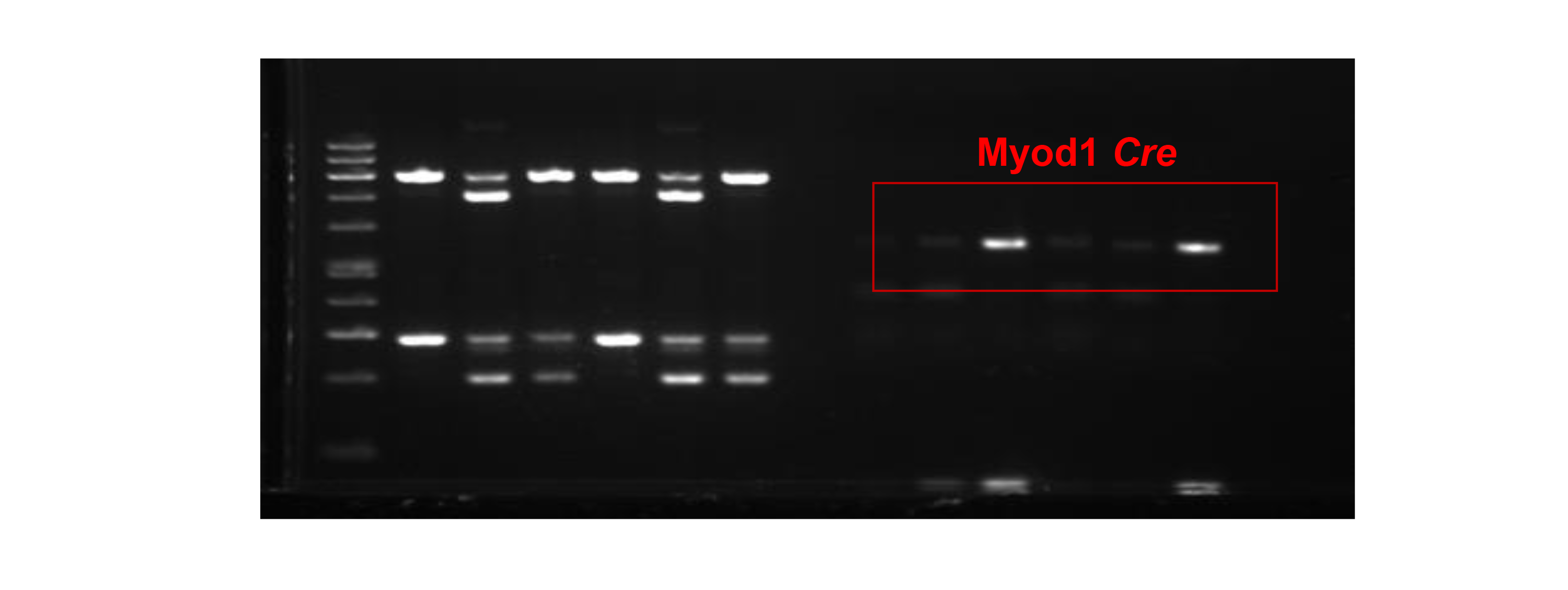

Supplement: Figure 1—figure supplement 1—source data 1. [file elife-98175-fig1-figsupp1-data1.zip › Fig 1-supplement 1B source data 1/Figure1-supplement 1B Myod1 Cre.tif]

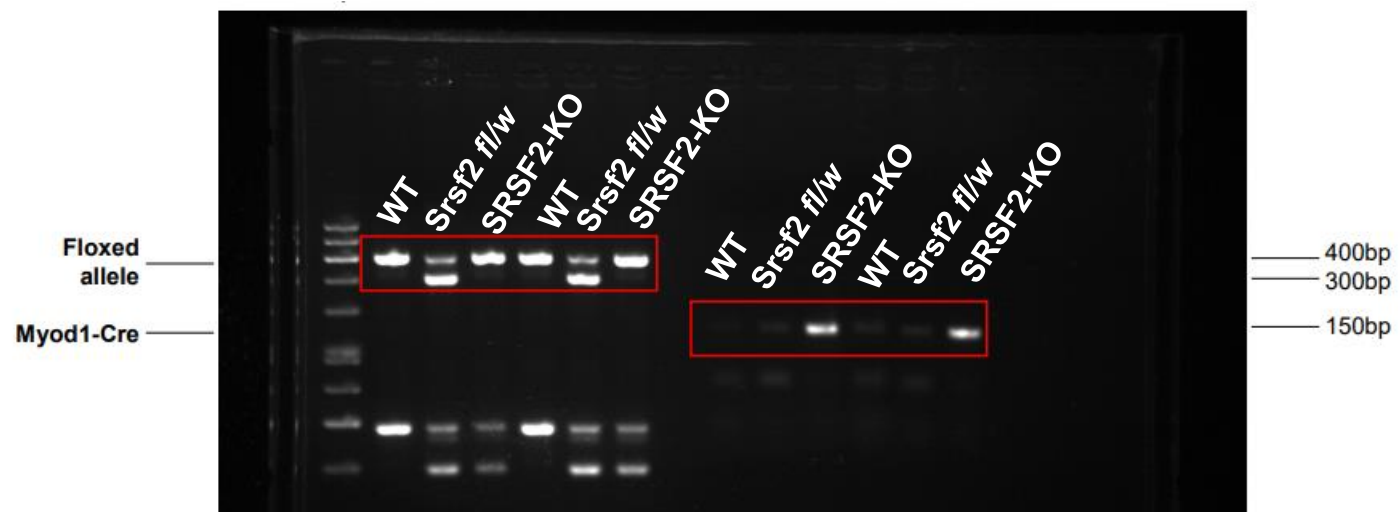

Figure 1— figure supplement 1

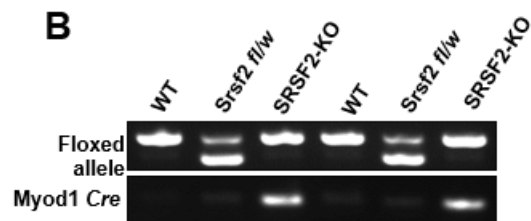

Supplement: Figure 1—figure supplement 1—source data 2. [file elife-98175-fig1-figsupp1-data2.zip › Fig.1-supplement 1B source data 2/Figure1-supplement 1B.pdf]

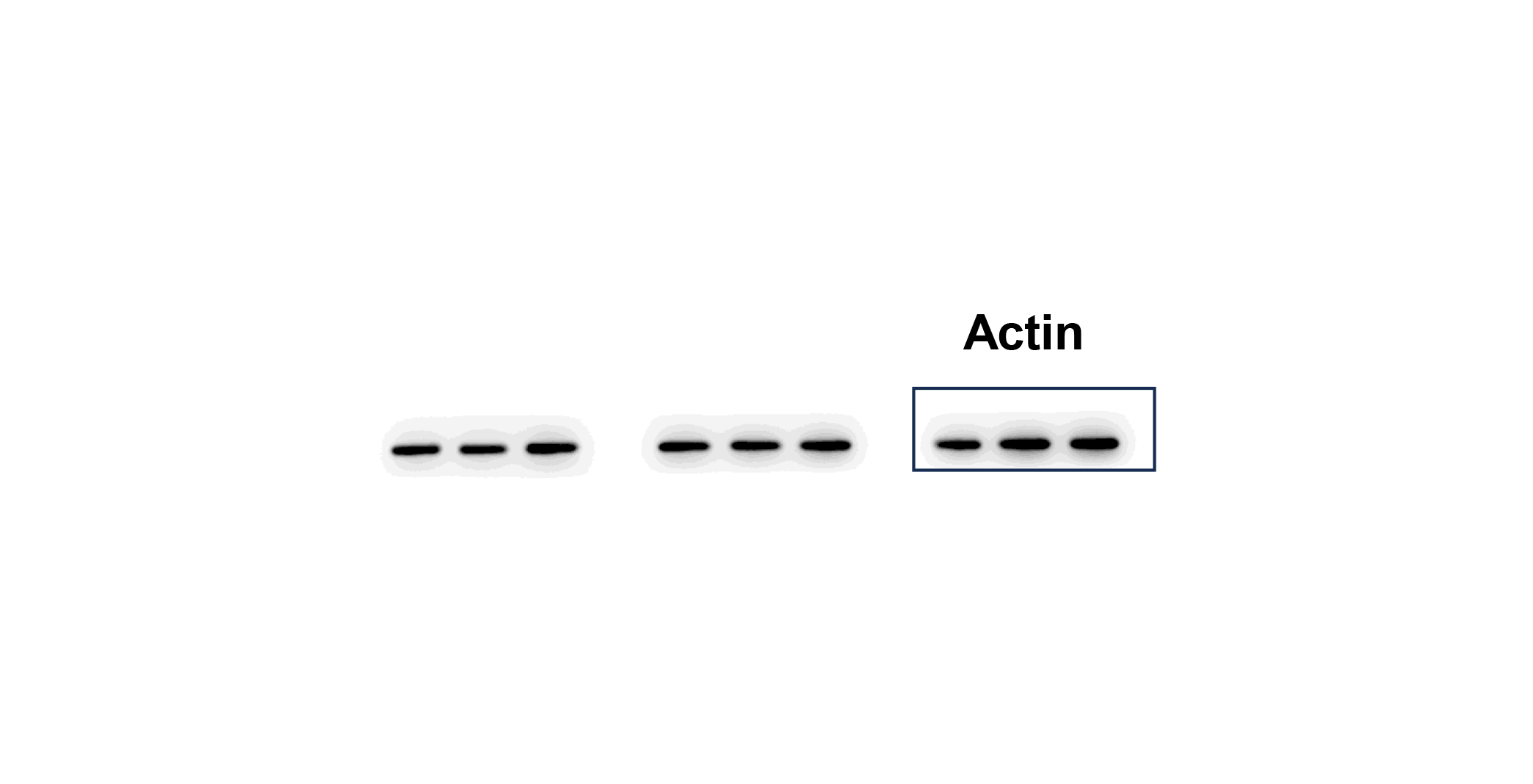

Supplement: Figure 7—source data 1. [file elife-98175-fig7-data1.zip › Figure 7—source data 1/Figure 7C-Aurka- knockdown-Actin.tif]

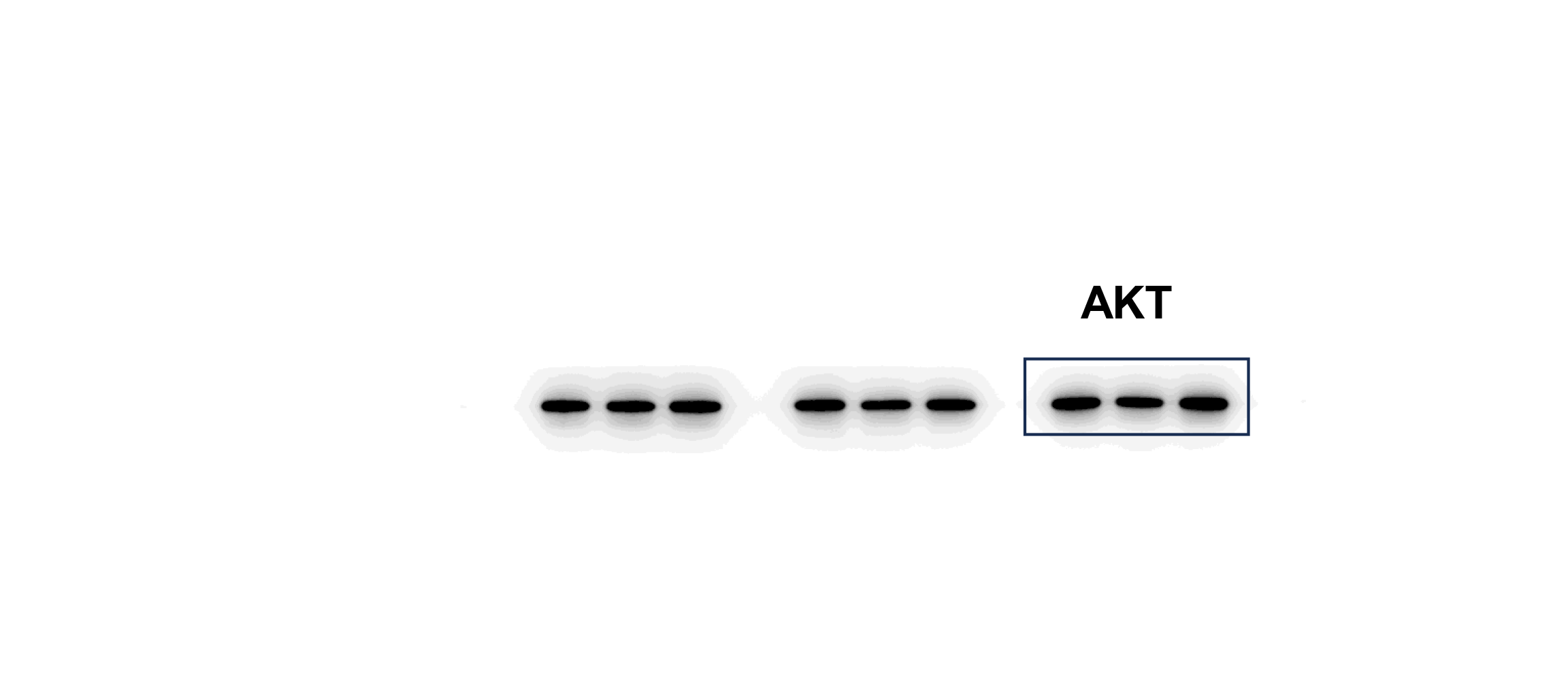

Supplement: Figure 7—source data 1. [file elife-98175-fig7-data1.zip › Figure 7—source data 1/Figure 7C-Aurka- knockdown-AKT.tif]

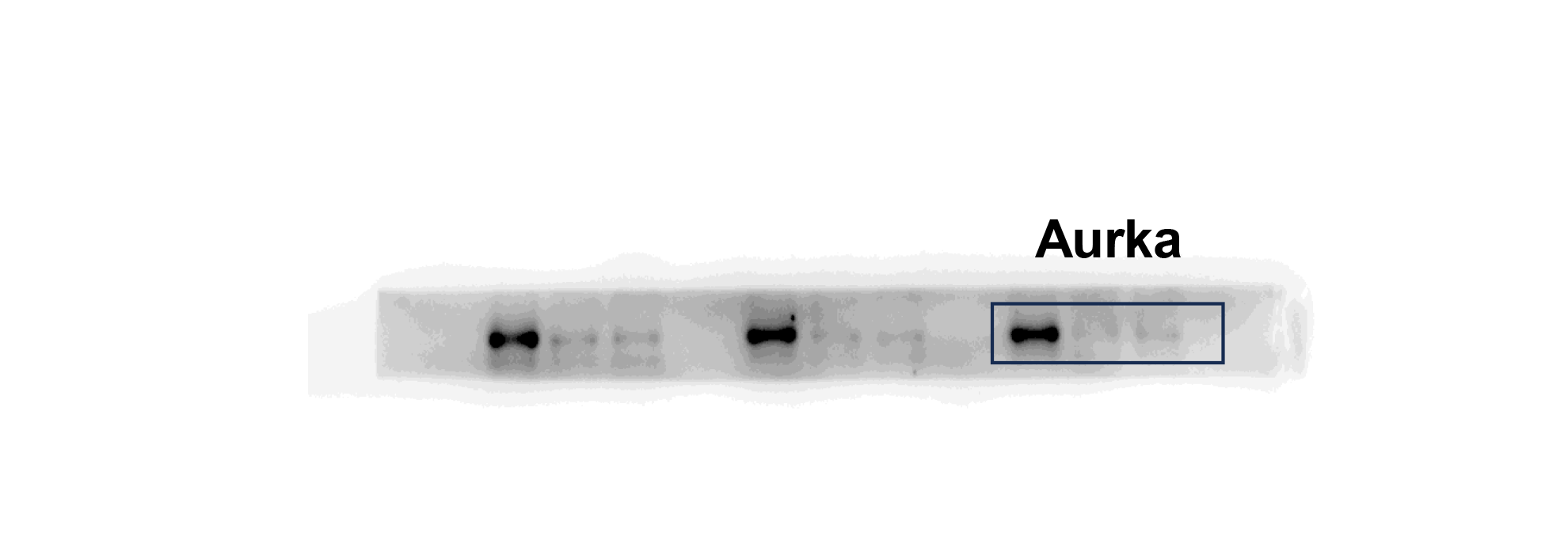

Supplement: Figure 7—source data 1. [file elife-98175-fig7-data1.zip › Figure 7—source data 1/Figure 7C-Aurka- knockdown-Aurka.tif]

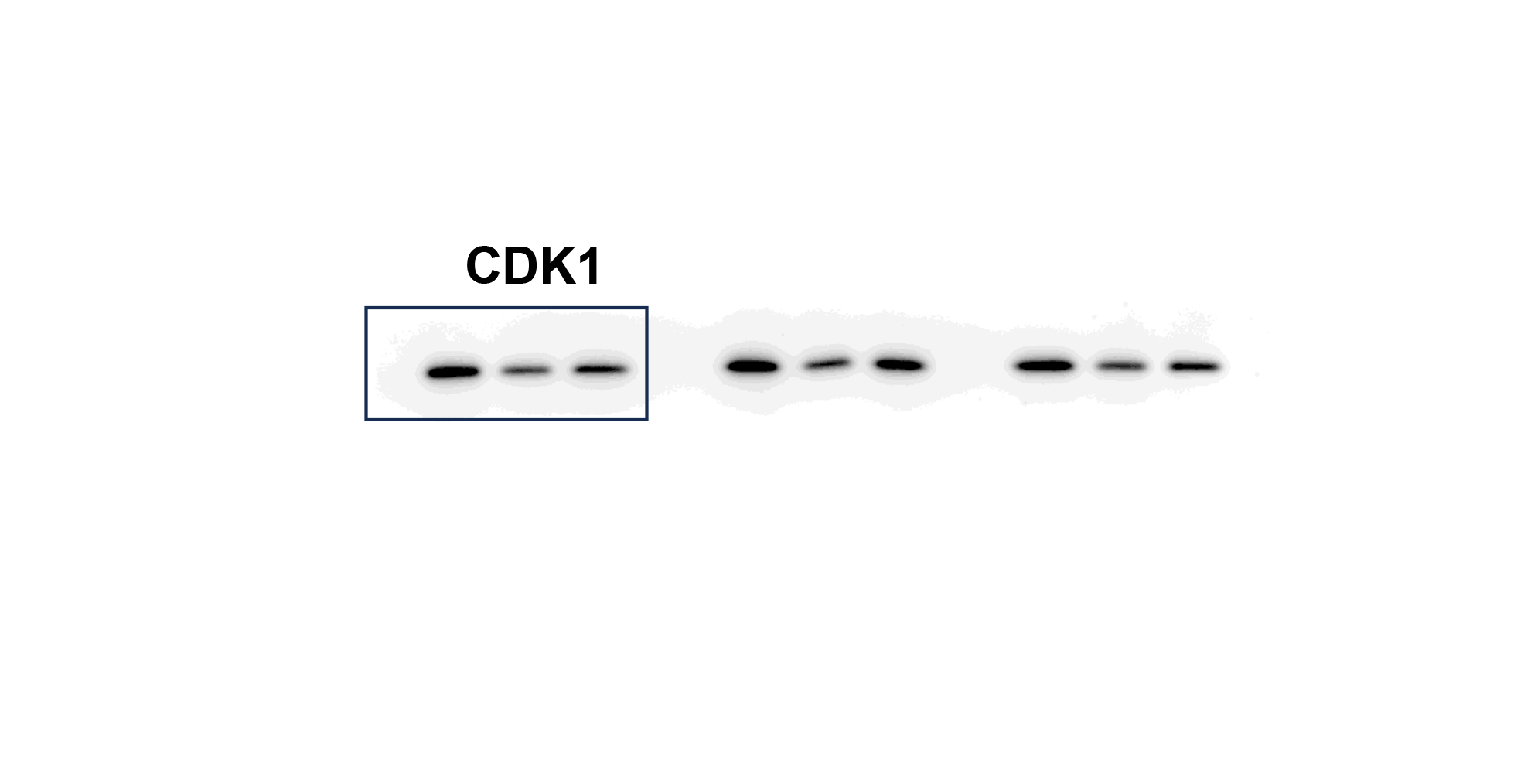

Supplement: Figure 7—source data 1. [file elife-98175-fig7-data1.zip › Figure 7—source data 1/Figure 7C-Aurka- knockdown-CDK1.tif]

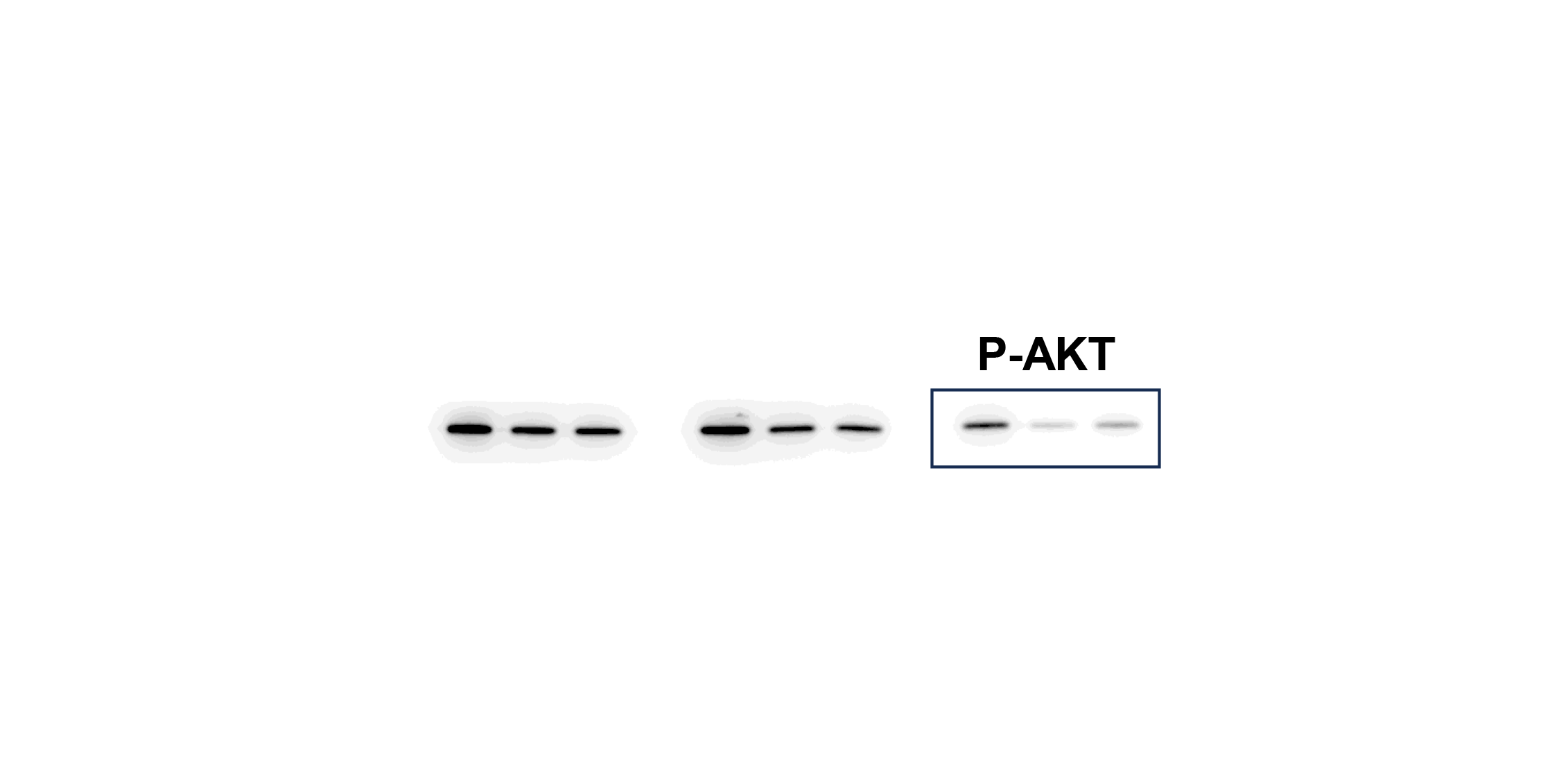

Supplement: Figure 7—source data 1. [file elife-98175-fig7-data1.zip › Figure 7—source data 1/Figure 7C-Aurka- knockdown-p-AKT.tif]

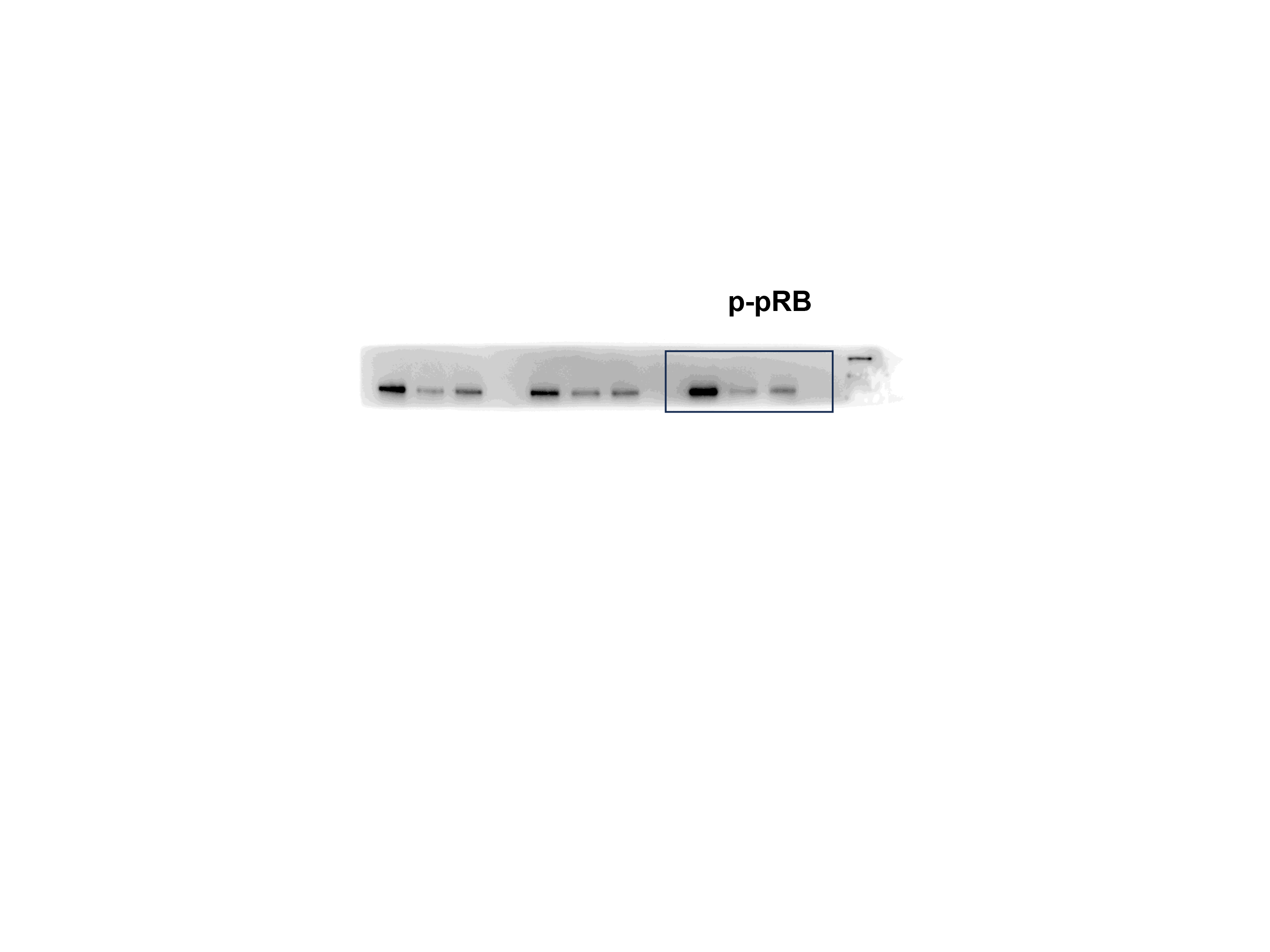

Supplement: Figure 7—source data 1. [file elife-98175-fig7-data1.zip › Figure 7—source data 1/Figure 7C-Aurka- knockdown-p-pRB.tif]

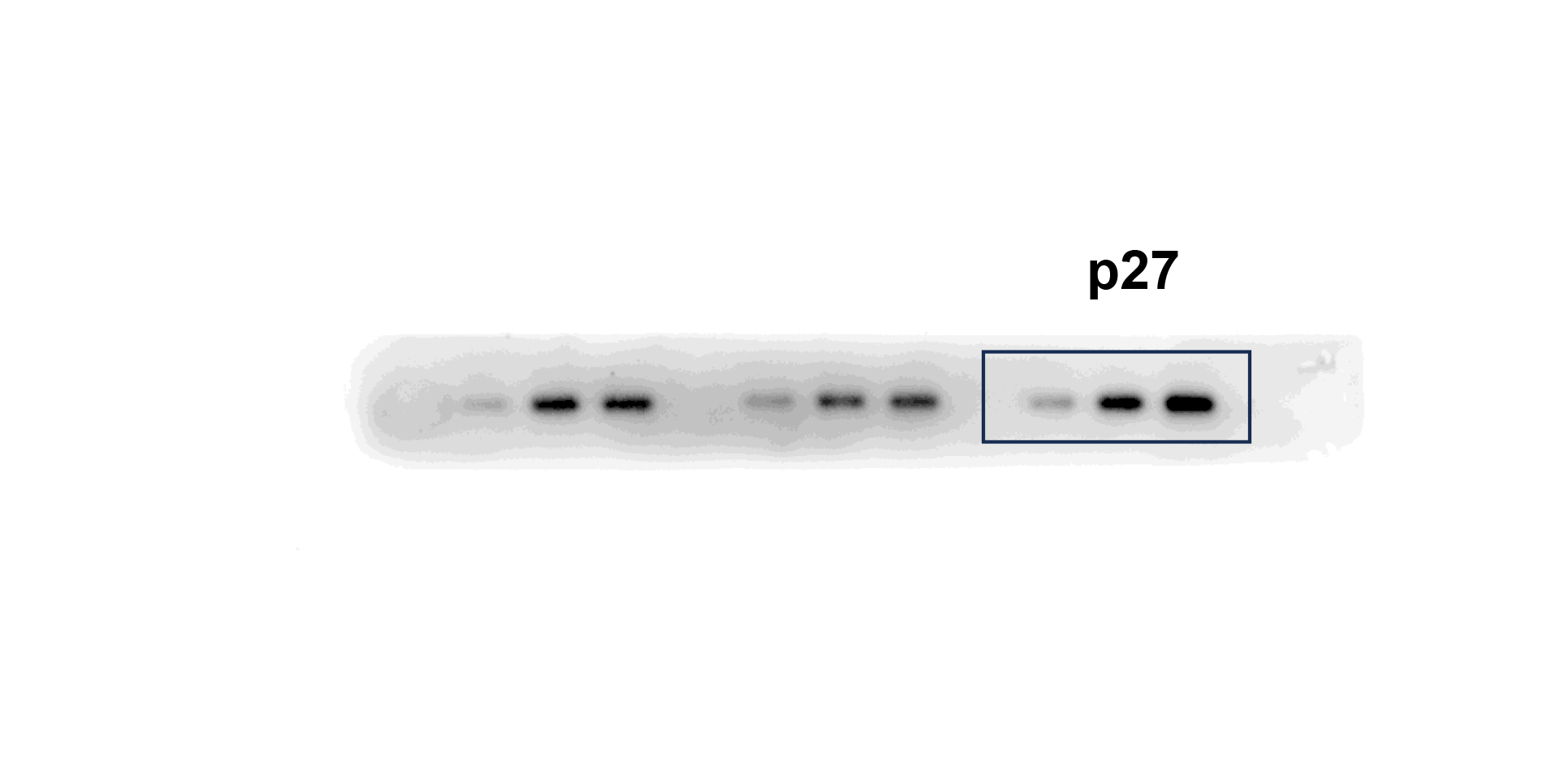

Supplement: Figure 7—source data 1. [file elife-98175-fig7-data1.zip › Figure 7—source data 1/Figure 7C-Aurka- knockdown-p27.tif]

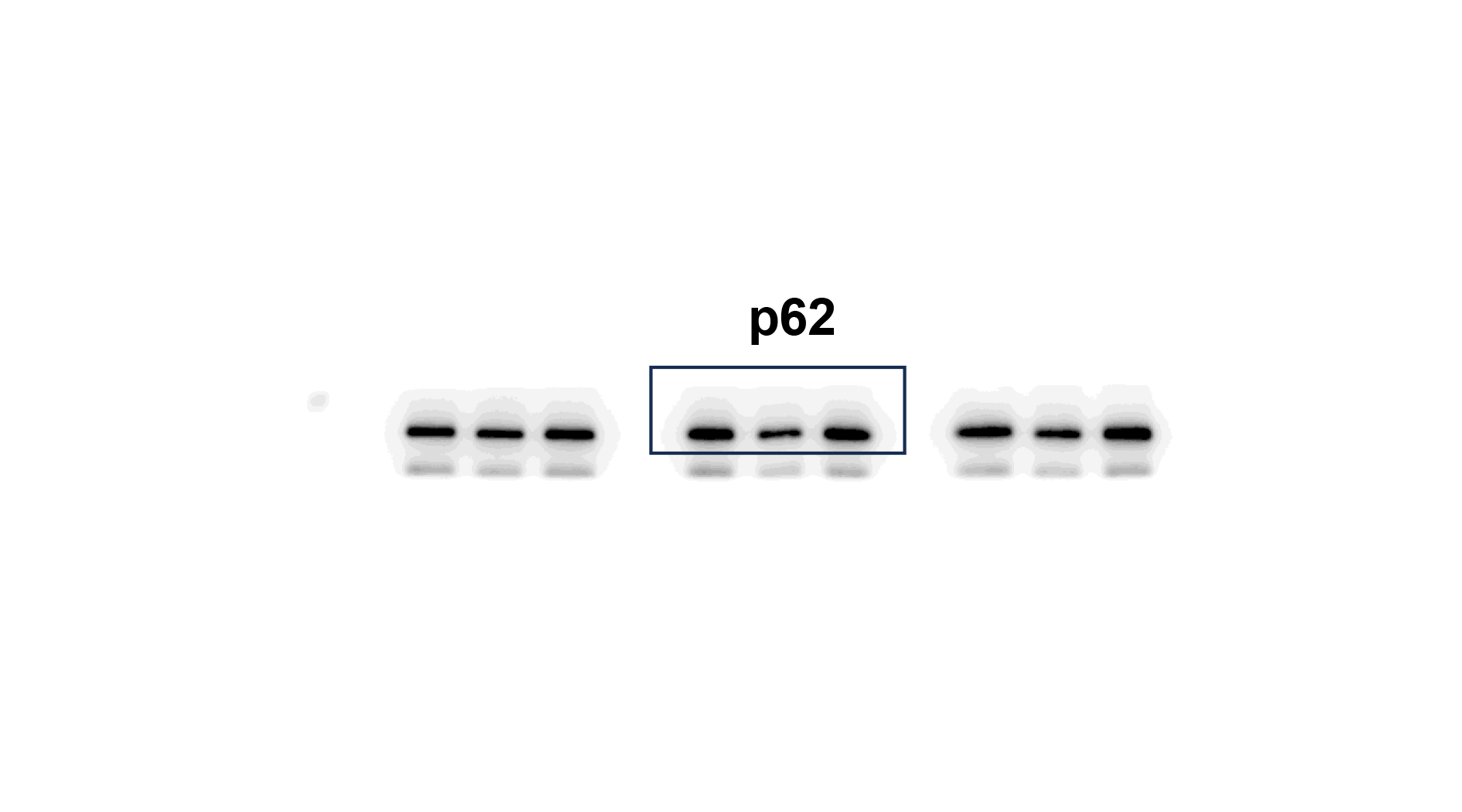

Supplement: Figure 7—source data 1. [file elife-98175-fig7-data1.zip › Figure 7—source data 1/Figure 7C-Aurka- knockdown-p62.tif]

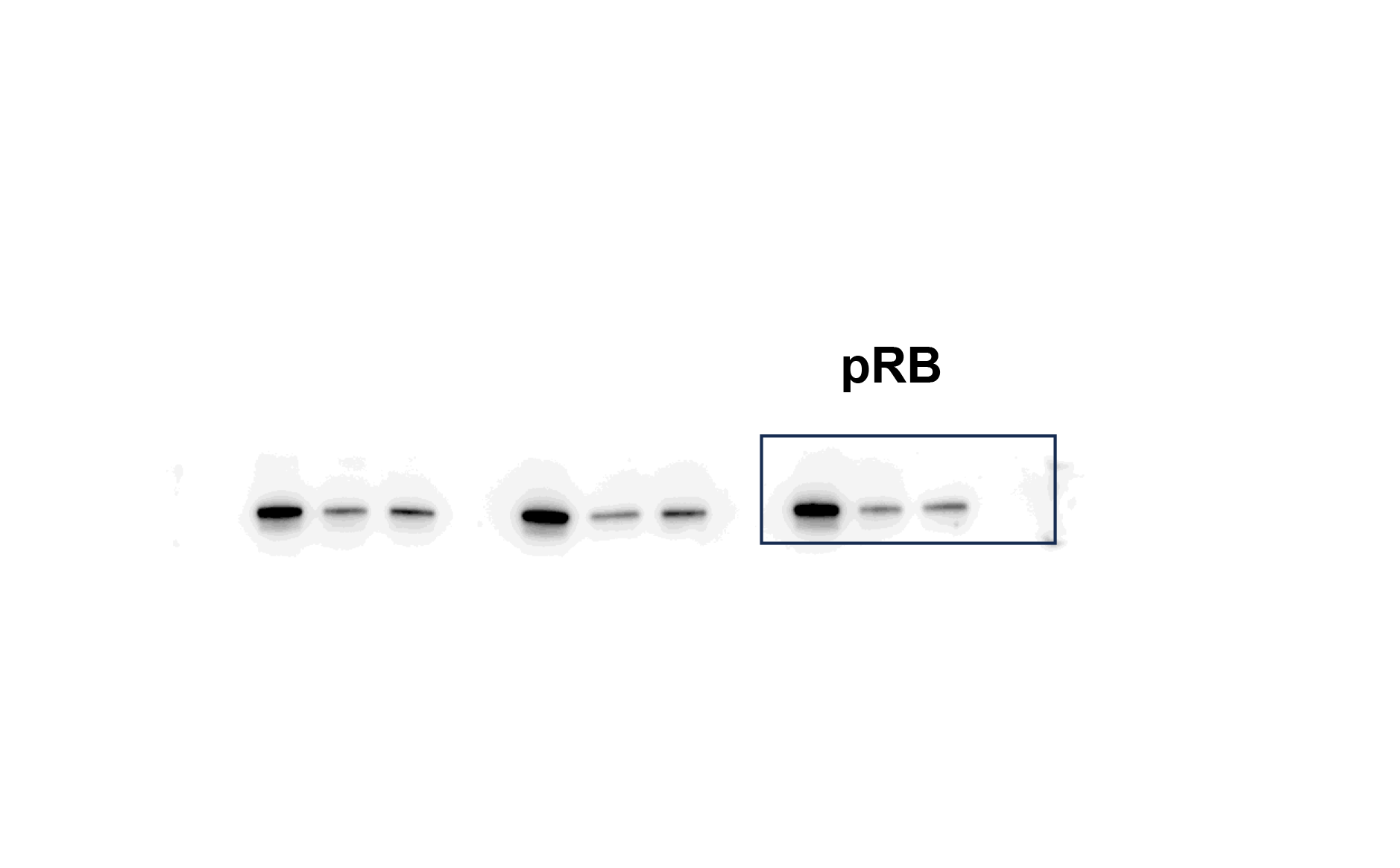

Supplement: Figure 7—source data 1. [file elife-98175-fig7-data1.zip › Figure 7—source data 1/Figure 7C-Aurka- knockdown-pRB.tif]

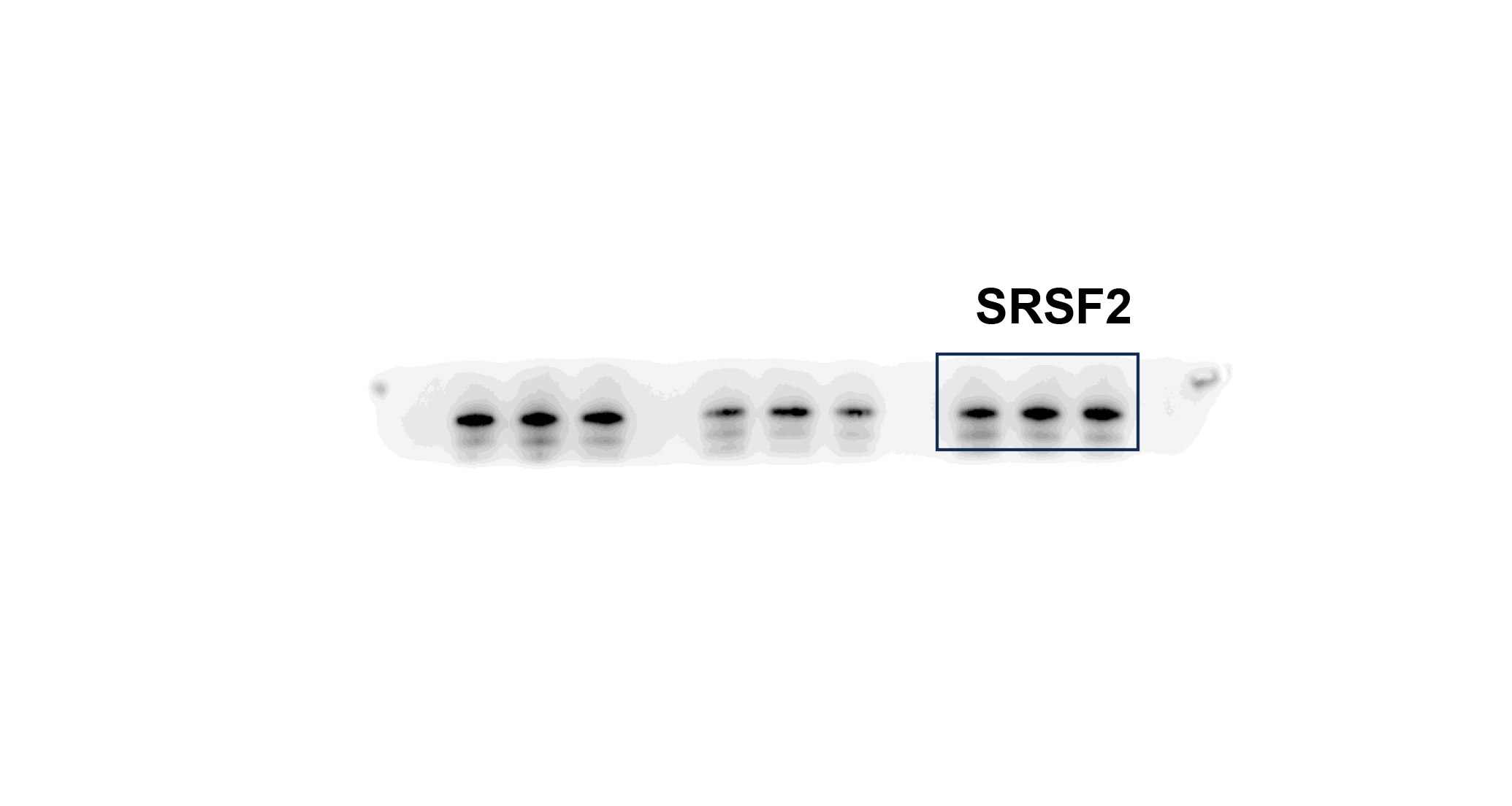

Supplement: Figure 7—source data 1. [file elife-98175-fig7-data1.zip › Figure 7—source data 1/Figure 7C-Aurka- knockdown-SRSF2.tif]

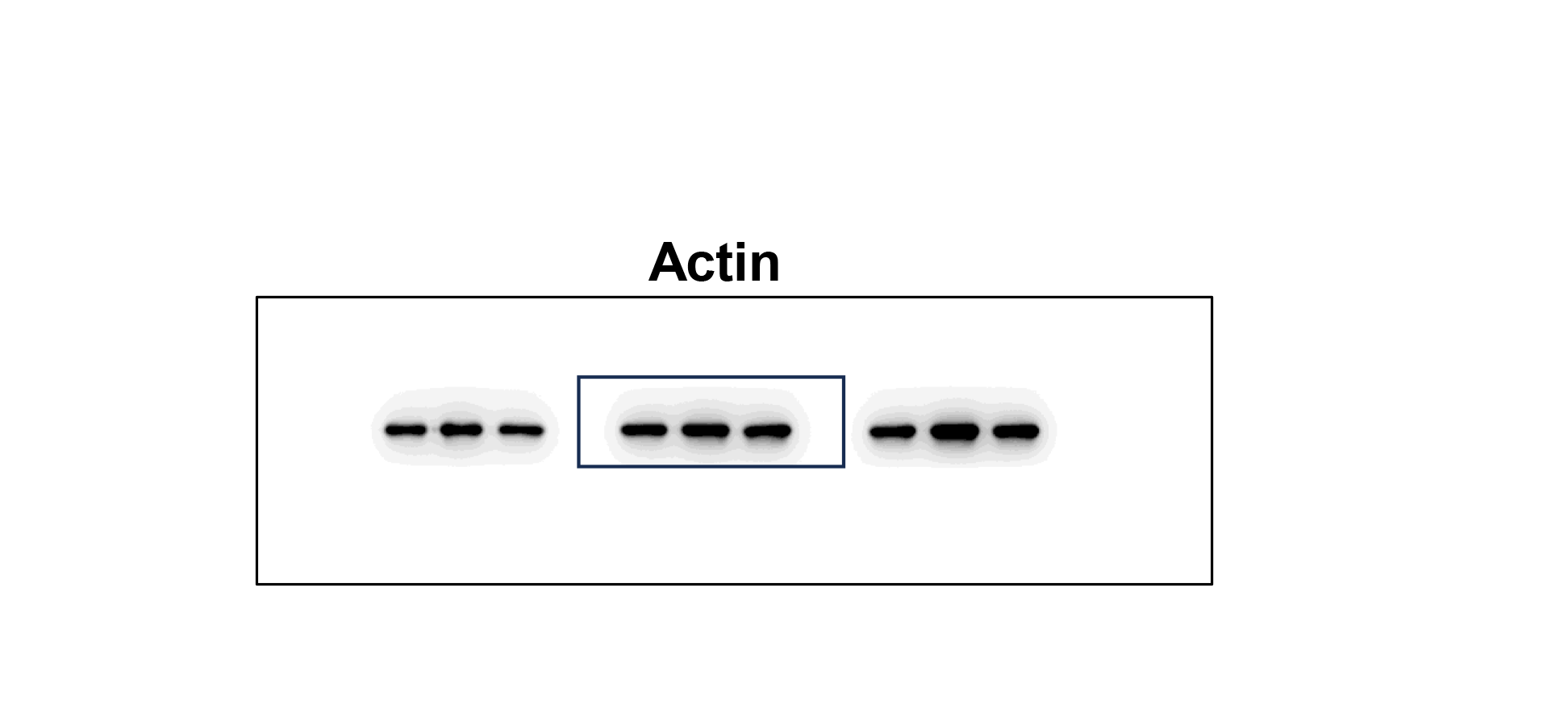

Supplement: Figure 7—source data 1. [file elife-98175-fig7-data1.zip › Figure 7—source data 1/Figure 7C-SRSF2 knockdown-Actin.tif]

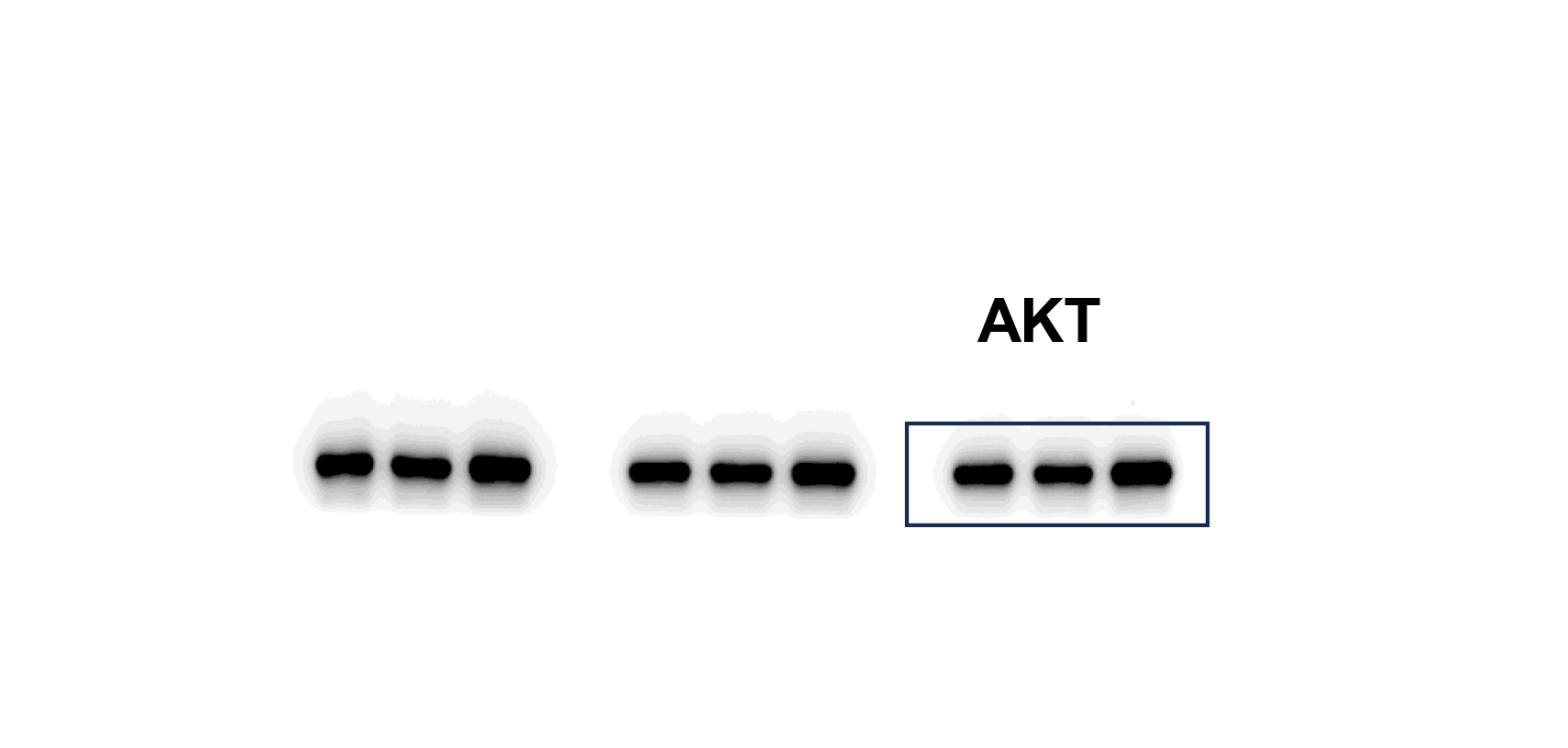

Supplement: Figure 7—source data 1. [file elife-98175-fig7-data1.zip › Figure 7—source data 1/Figure 7C-SRSF2 knockdown-AKT.tif]

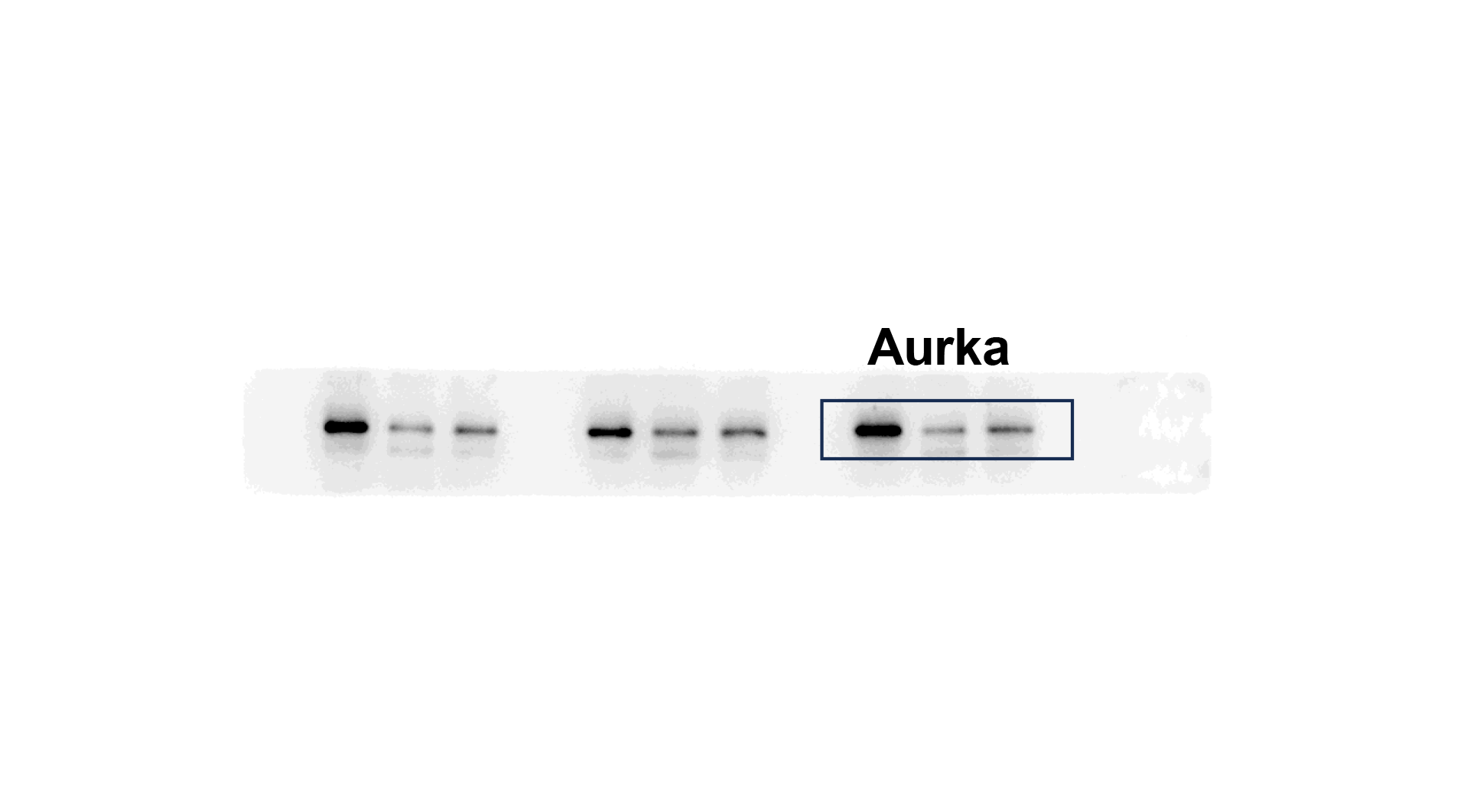

Supplement: Figure 7—source data 1. [file elife-98175-fig7-data1.zip › Figure 7—source data 1/Figure 7C-SRSF2 knockdown-Aurka.tif]

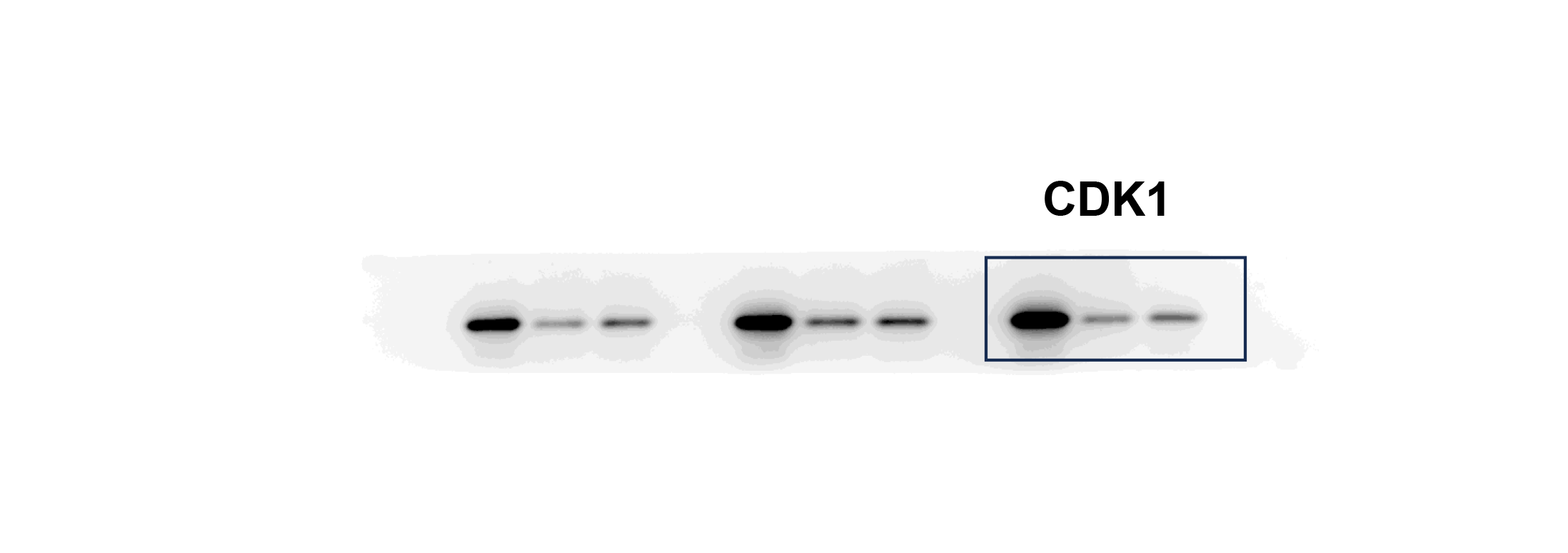

Supplement: Figure 7—source data 1. [file elife-98175-fig7-data1.zip › Figure 7—source data 1/Figure 7C-SRSF2 knockdown-CDK1.tif]

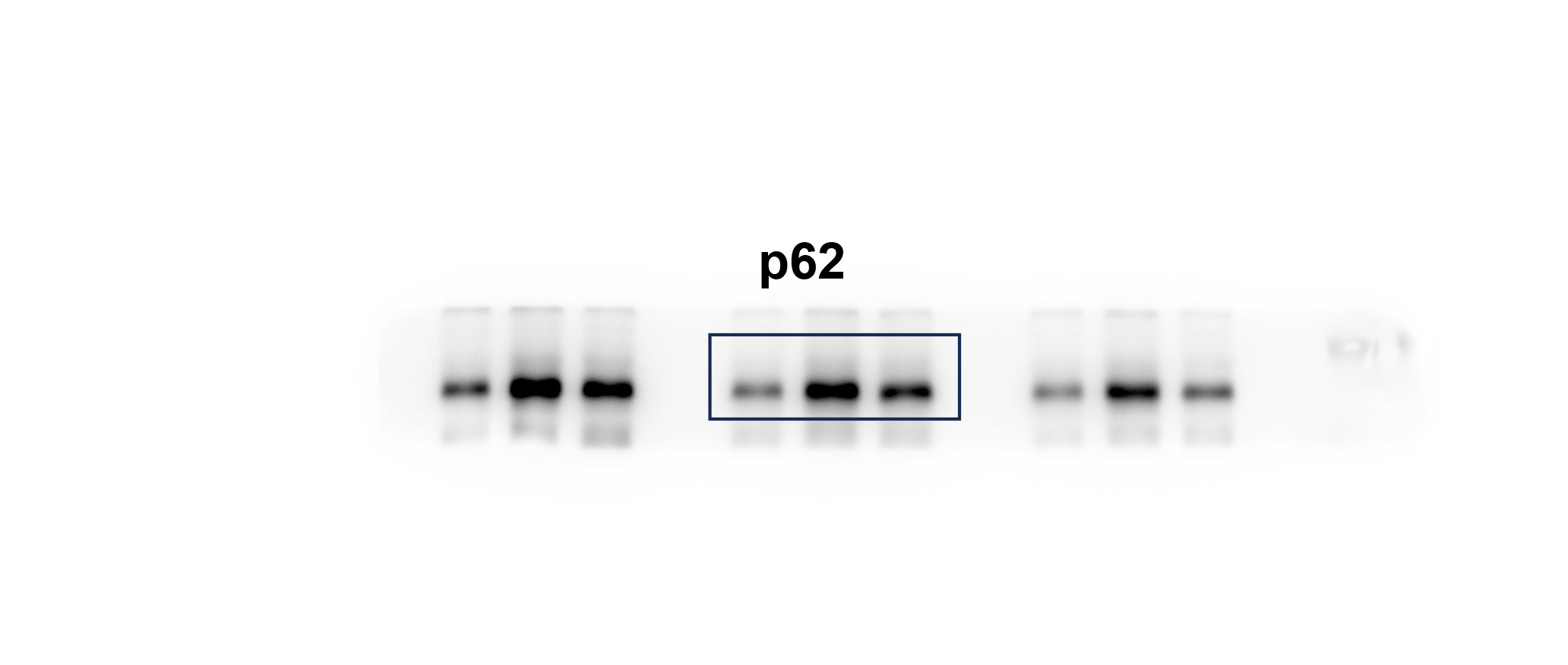

Supplement: Figure 7—source data 1. [file elife-98175-fig7-data1.zip › Figure 7—source data 1/Figure 7C-SRSF2 knockdown-p-62.tif]

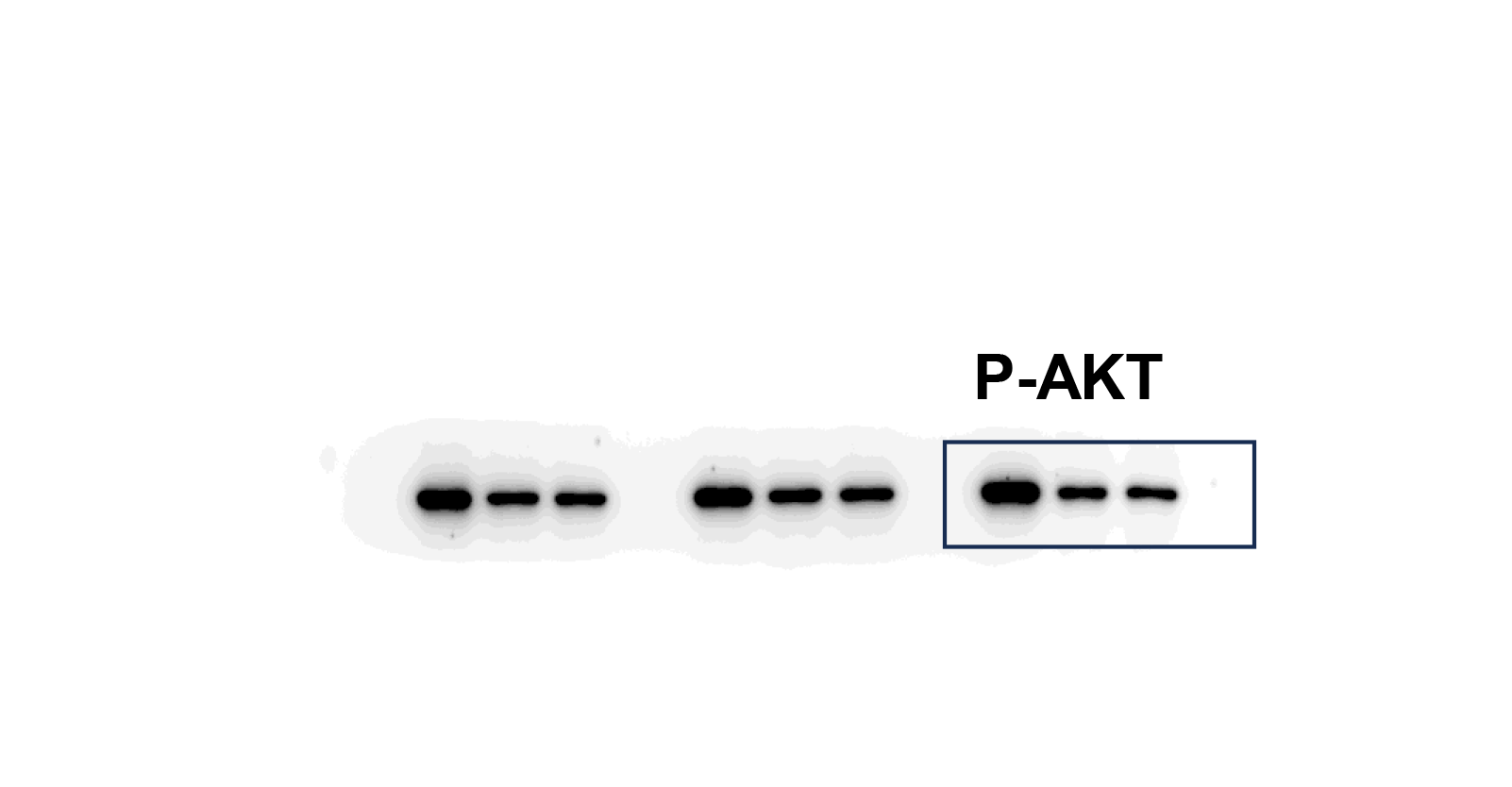

Supplement: Figure 7—source data 1. [file elife-98175-fig7-data1.zip › Figure 7—source data 1/Figure 7C-SRSF2 knockdown-p-AKT.tif]

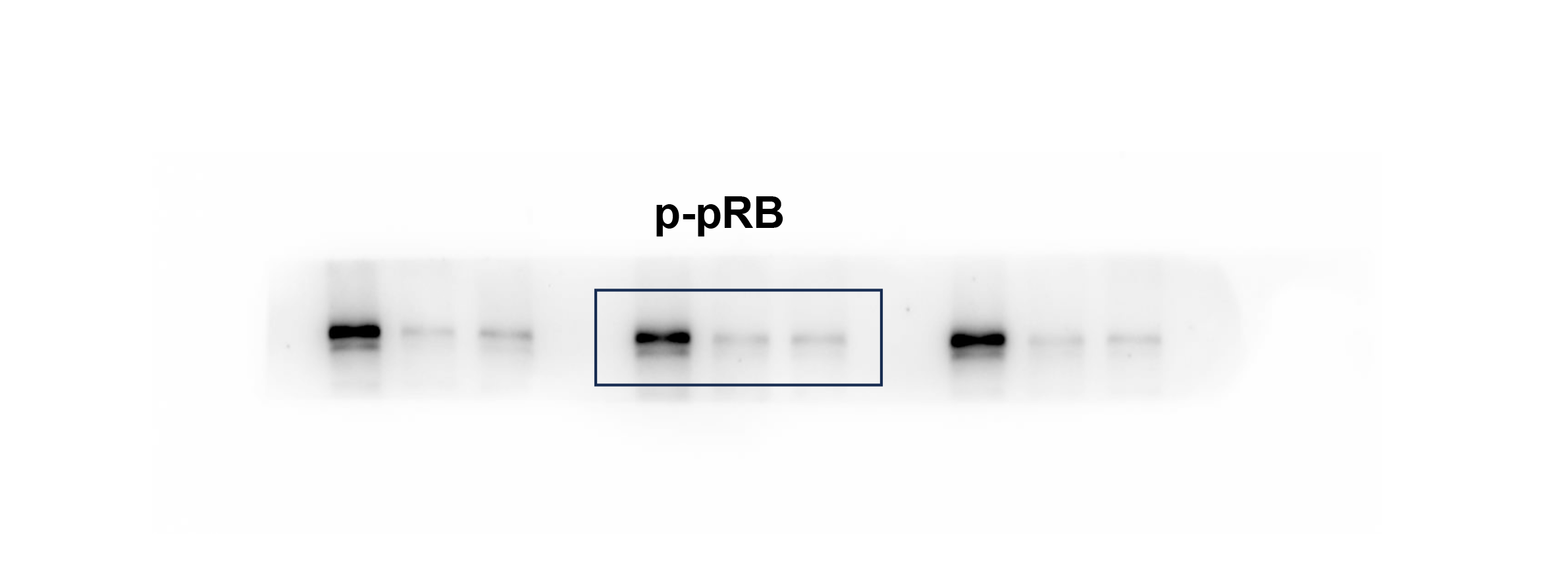

Supplement: Figure 7—source data 1. [file elife-98175-fig7-data1.zip › Figure 7—source data 1/Figure 7C-SRSF2 knockdown-p-pRB.tif]

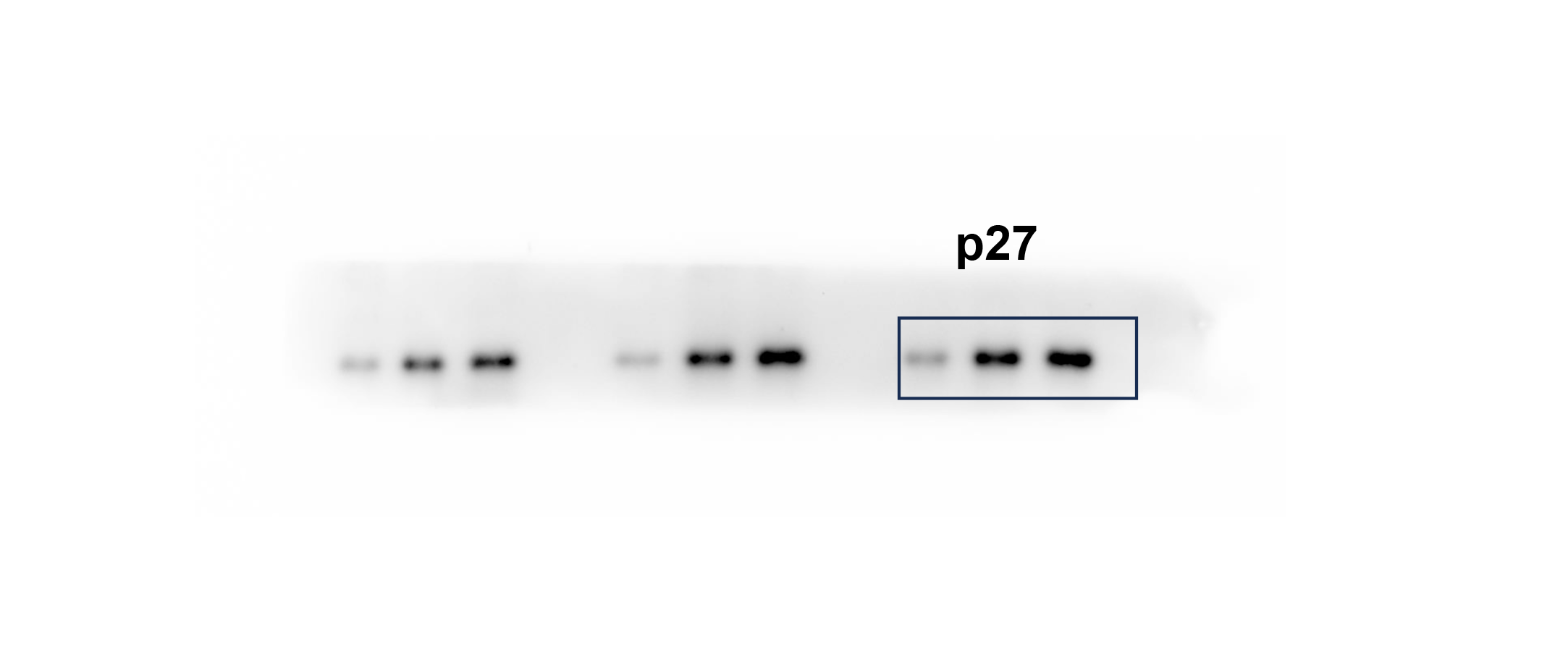

Supplement: Figure 7—source data 1. [file elife-98175-fig7-data1.zip › Figure 7—source data 1/Figure 7C-SRSF2 knockdown-p27.tif]

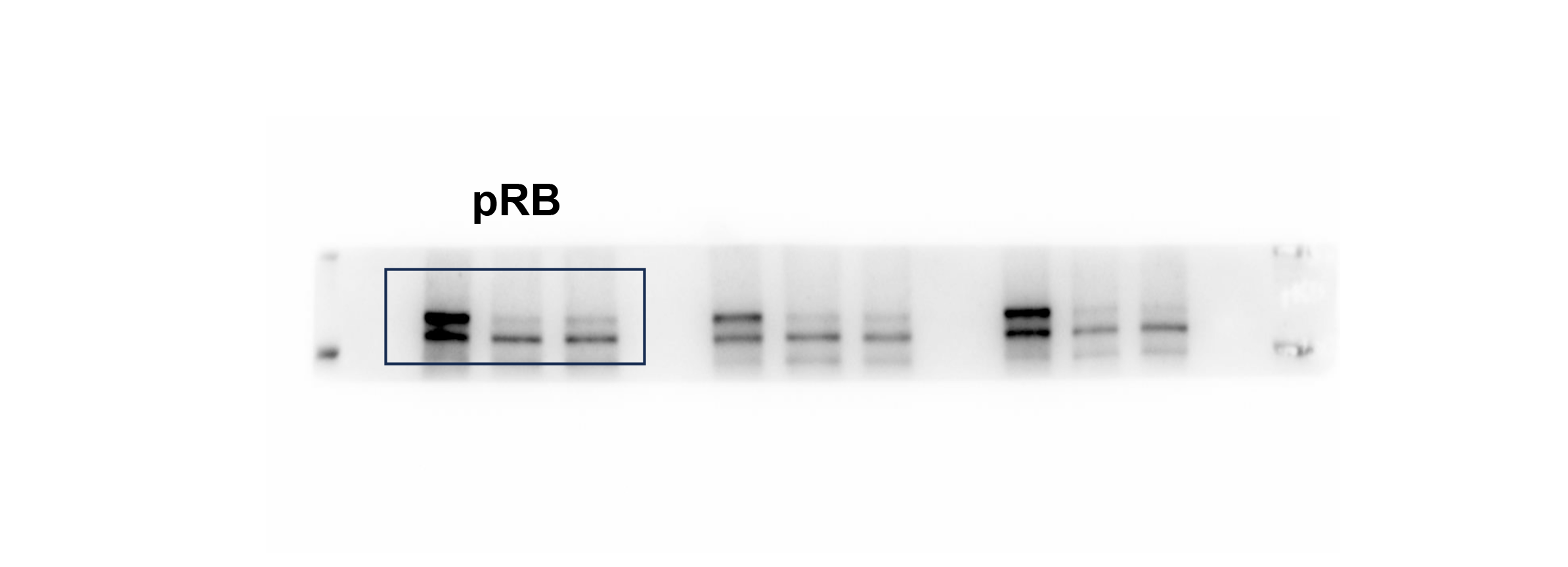

Supplement: Figure 7—source data 1. [file elife-98175-fig7-data1.zip › Figure 7—source data 1/Figure 7C-SRSF2 knockdown-pRB.tif]

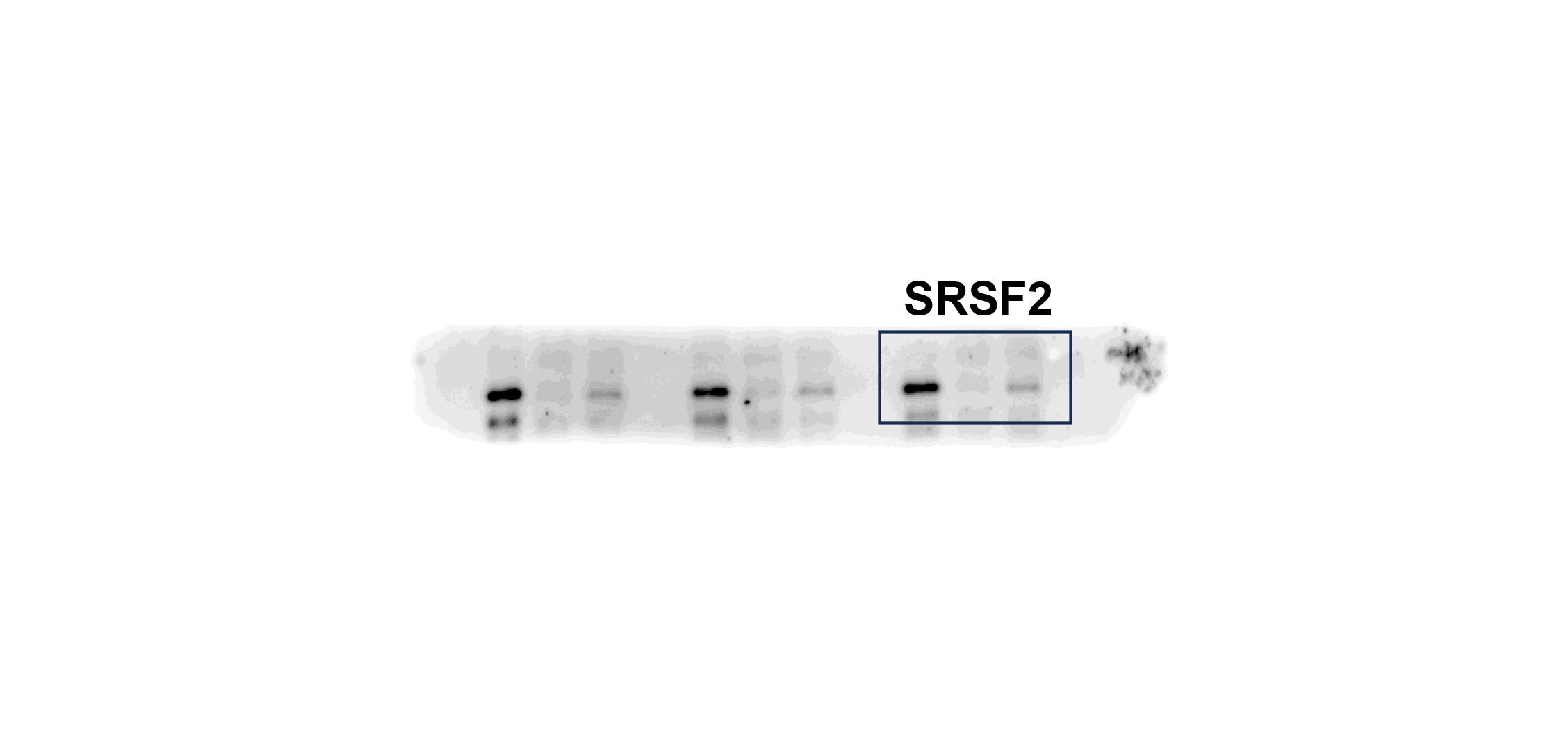

Supplement: Figure 7—source data 1. [file elife-98175-fig7-data1.zip › Figure 7—source data 1/Figure 7C-SRSF2 knockdown-SRSF2.tif]

**Figure 7C**

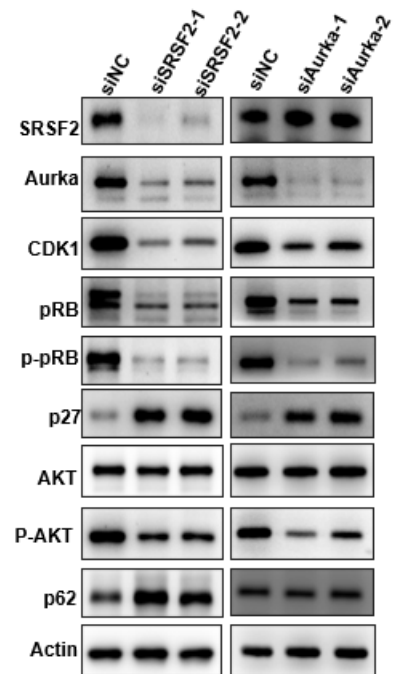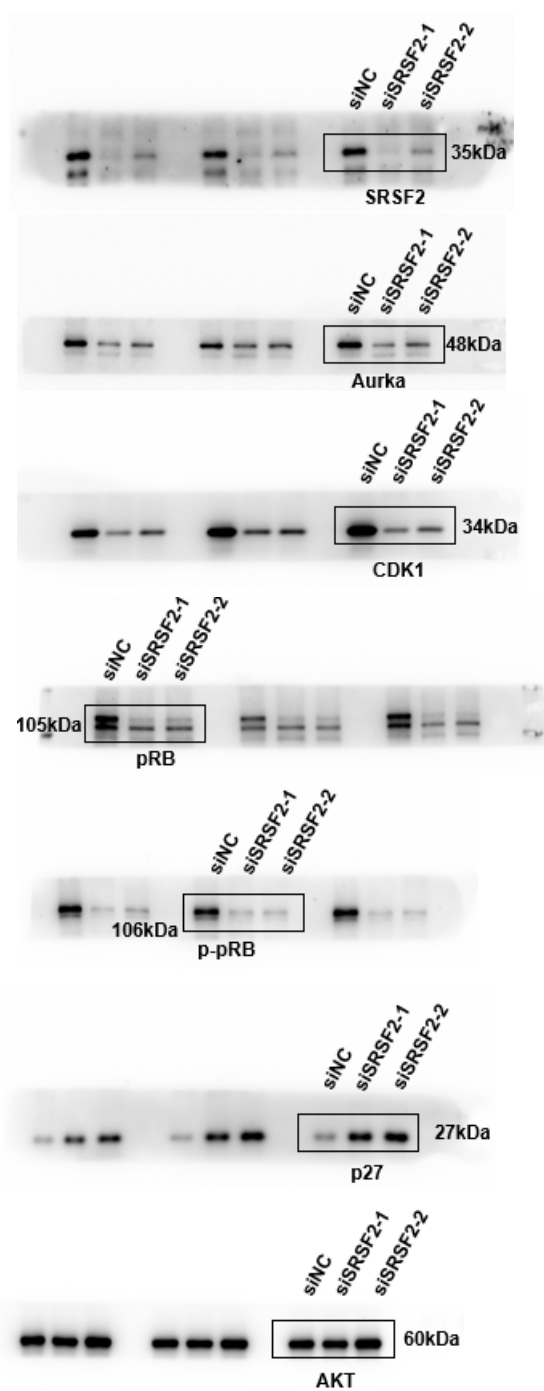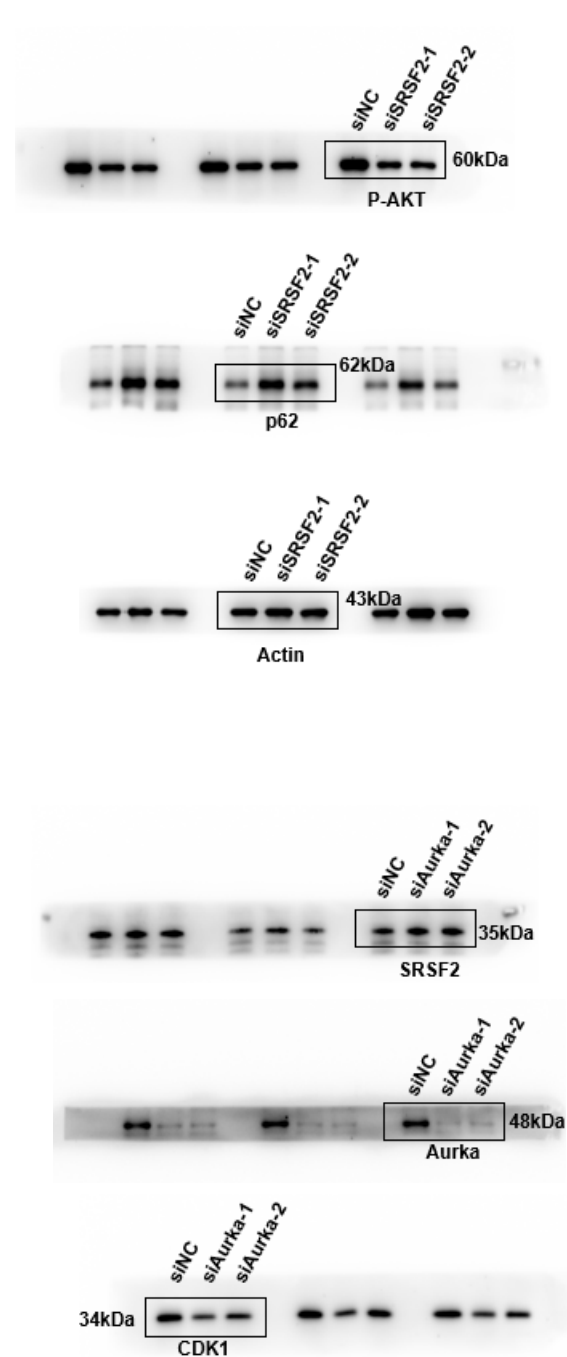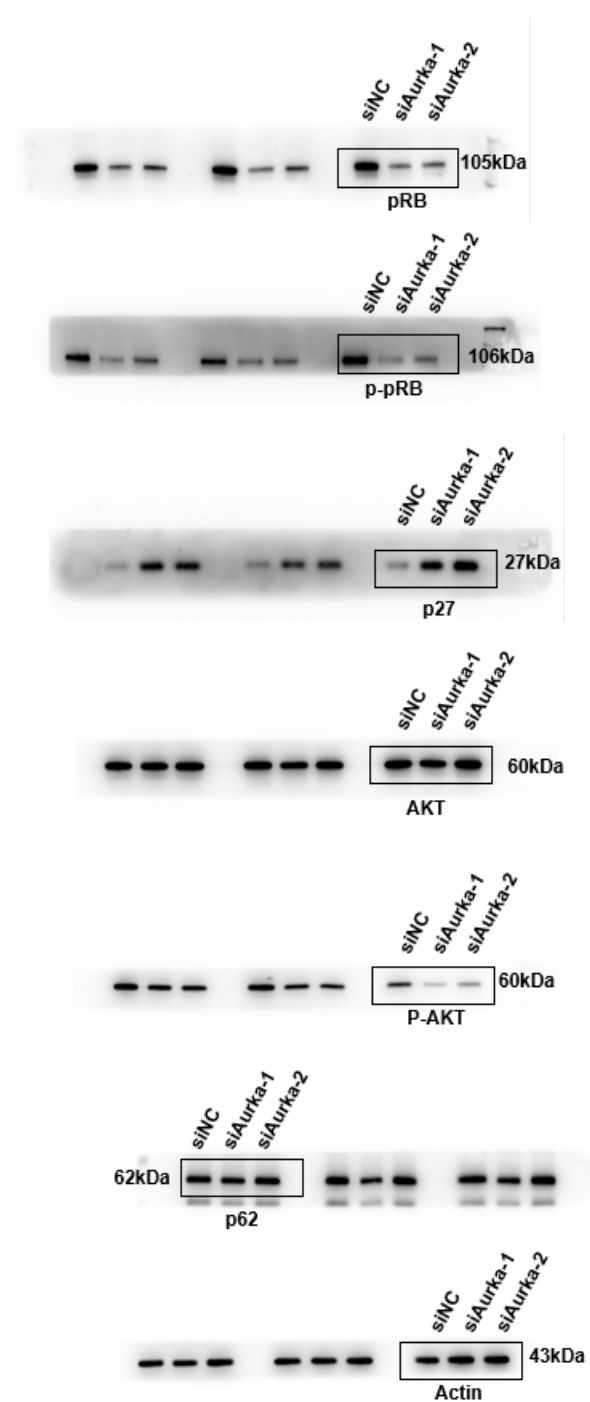

Supplement: Figure 7—source data 2. [file elife-98175-fig7-data2.zip › Figure 7—source data 2/Figure 7C.pdf]

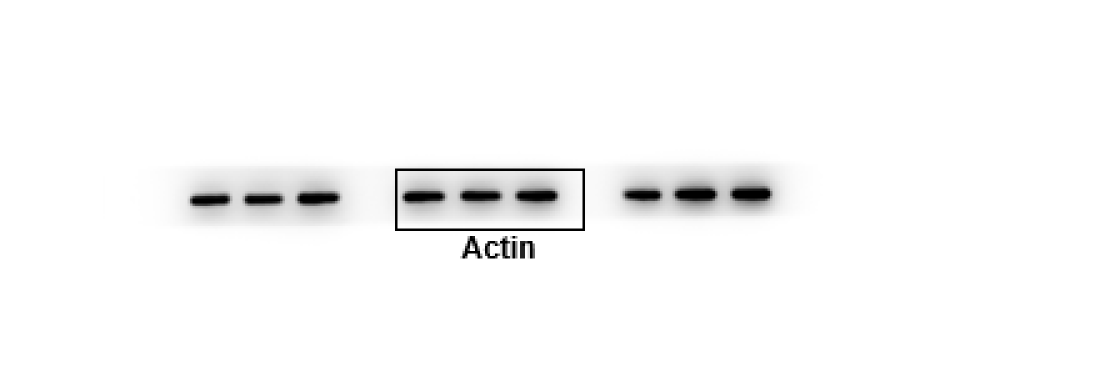

Supplement: Figure 7—source data 3. [file elife-98175-fig7-data3.zip › Figure 7—source data 3/Figure 7H-AurkaKD-Actin.tif]

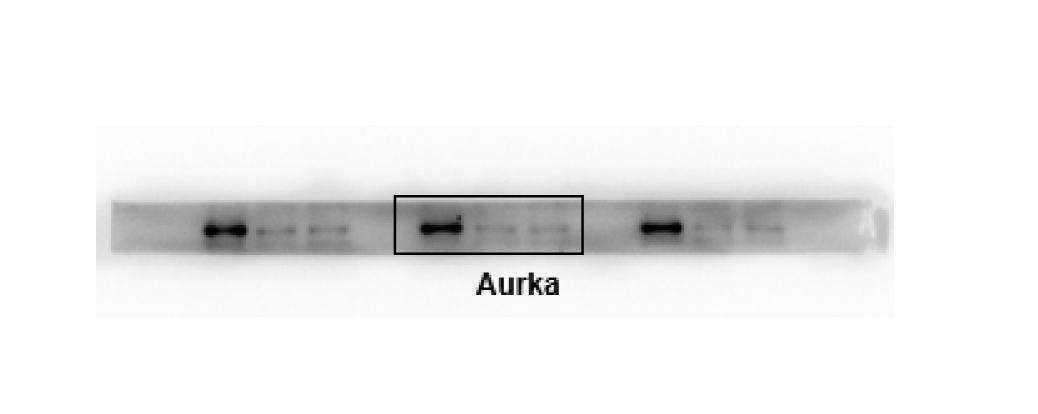

Supplement: Figure 7—source data 3. [file elife-98175-fig7-data3.zip › Figure 7—source data 3/Figure 7H-AurkaKD-Aurka.tif]

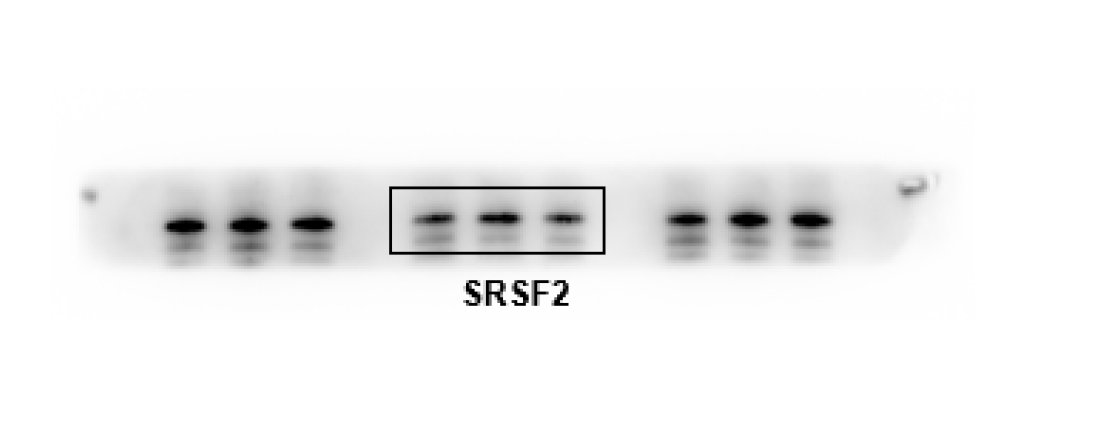

Supplement: Figure 7—source data 3. [file elife-98175-fig7-data3.zip › Figure 7—source data 3/Figure 7H-AurkaKD-SRSF2.tif]

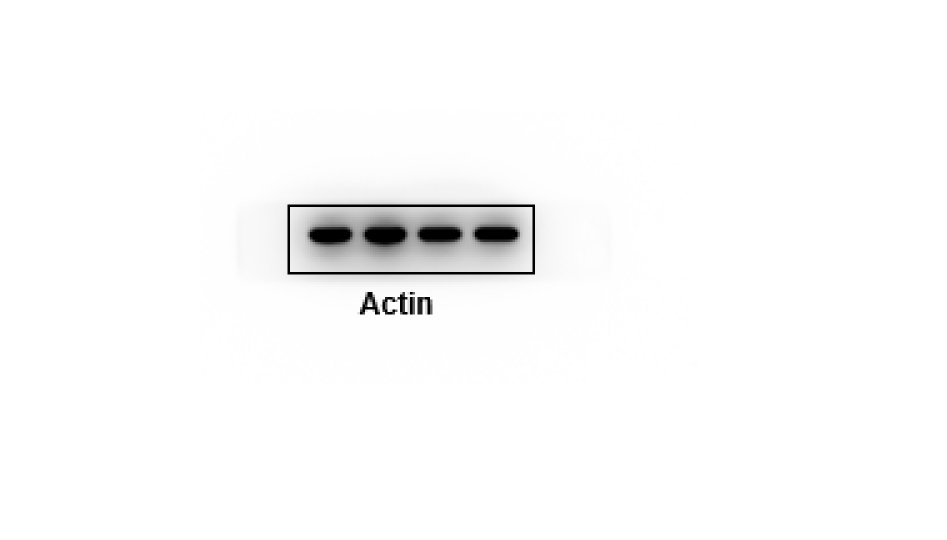

Supplement: Figure 7—source data 3. [file elife-98175-fig7-data3.zip › Figure 7—source data 3/Figure 7H-rescue-Actin.tif]

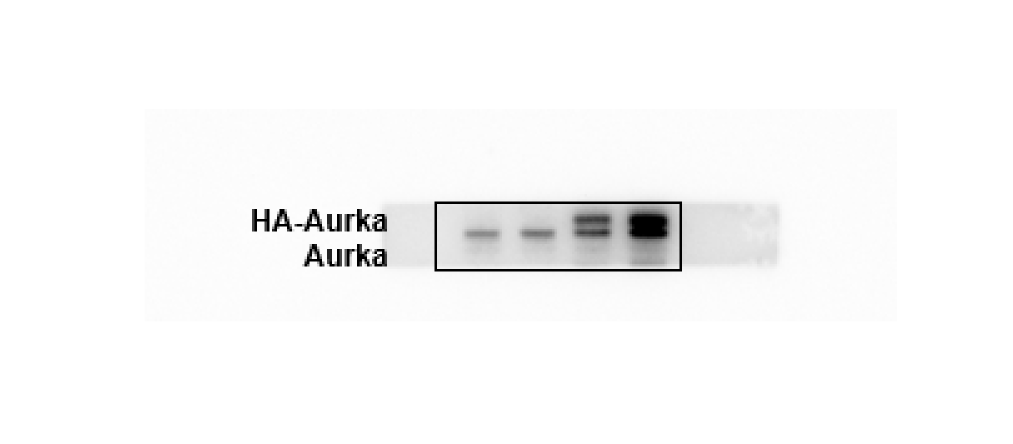

Supplement: Figure 7—source data 3. [file elife-98175-fig7-data3.zip › Figure 7—source data 3/Figure 7H-rescue-Aurka.tif]

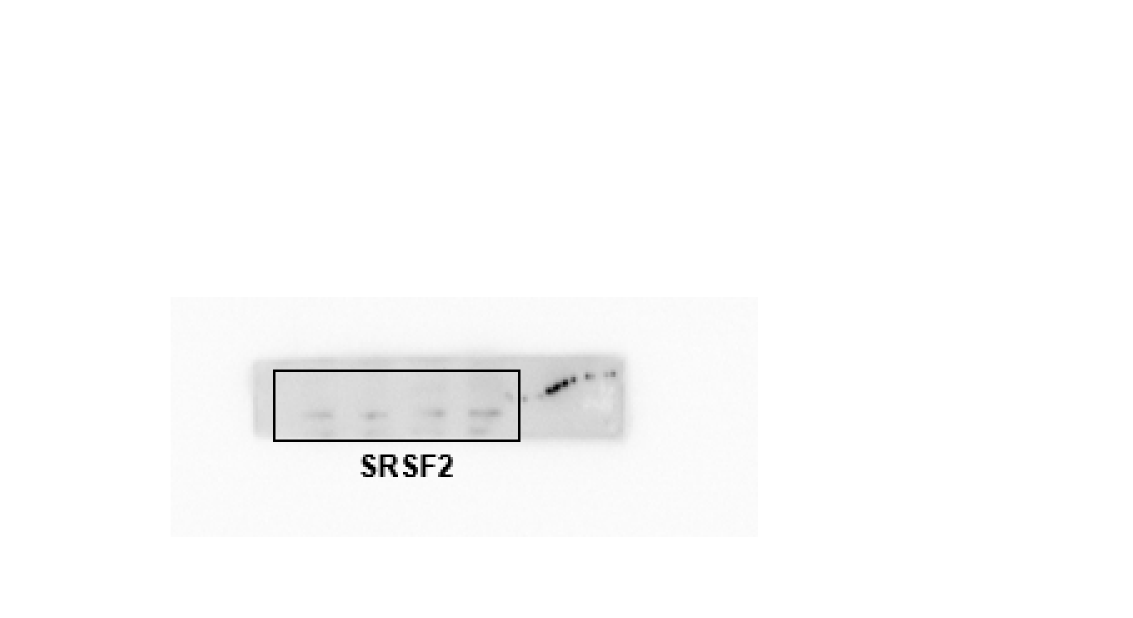

Supplement: Figure 7—source data 3. [file elife-98175-fig7-data3.zip › Figure 7—source data 3/Figure 7H-rescue-SRSF2.tif]

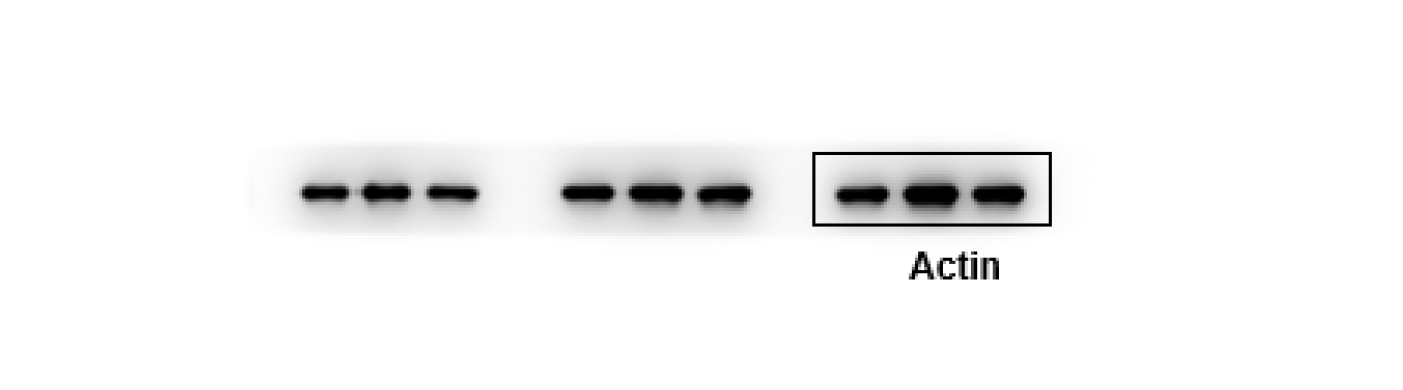

Supplement: Figure 7—source data 3. [file elife-98175-fig7-data3.zip › Figure 7—source data 3/Figure 7H-SRSF2KD-Actin.tif]

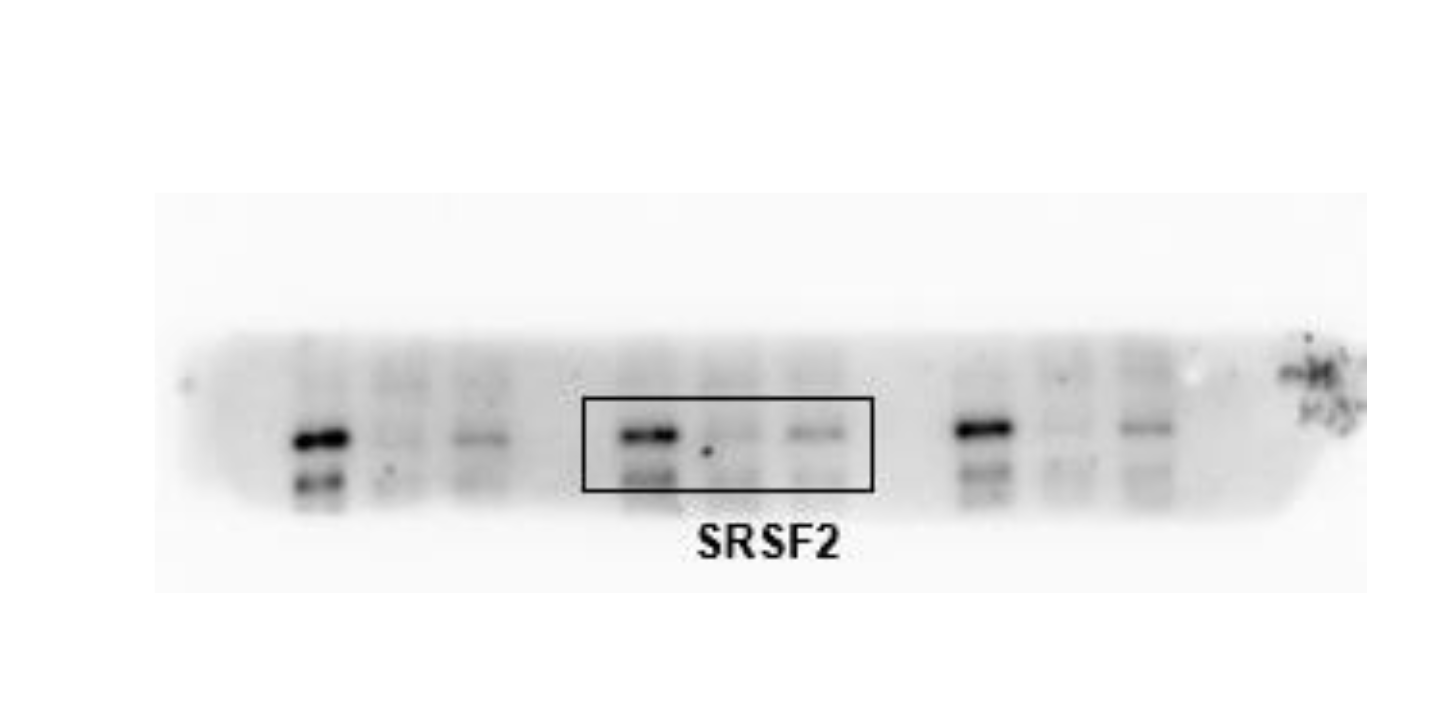

Supplement: Figure 7—source data 3. [file elife-98175-fig7-data3.zip › Figure 7—source data 3/Figure 7H-SRSF2KD-SRSF2.tif]

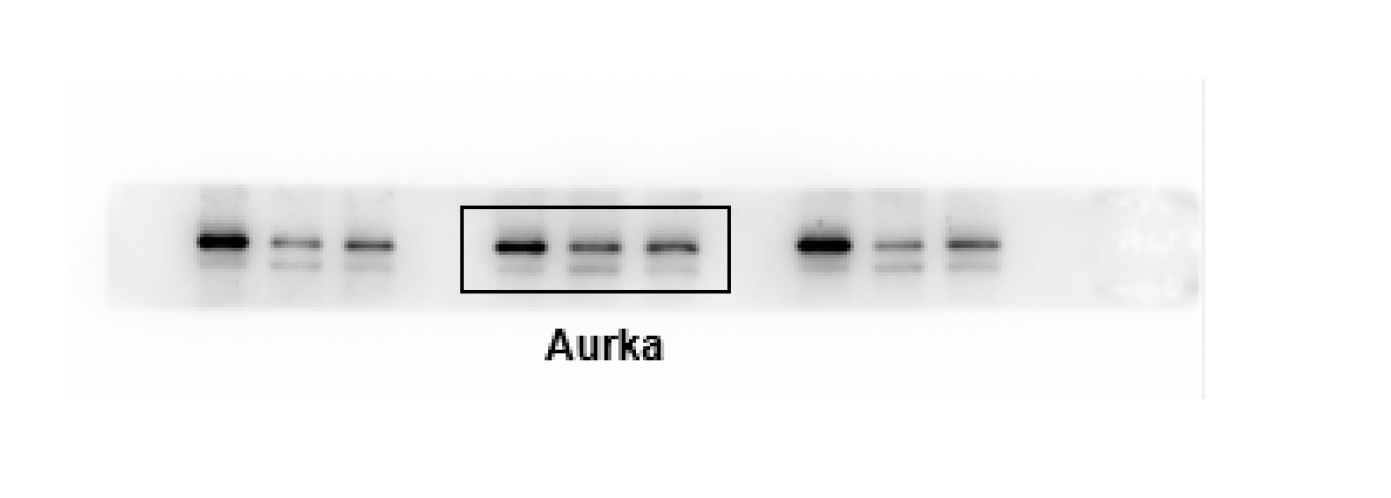

Supplement: Figure 7—source data 3. [file elife-98175-fig7-data3.zip › Figure 7—source data 3/Figure 7H-SRSFKD-Aurka.tif]

Figure 7H

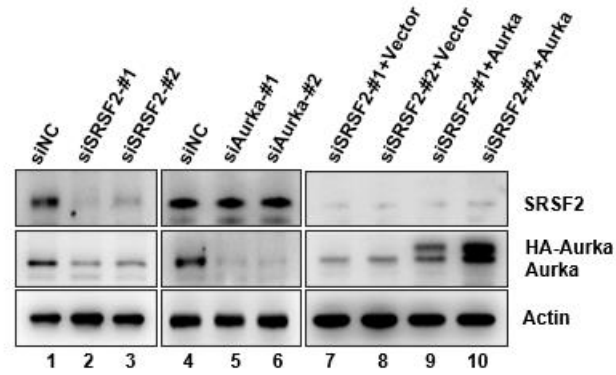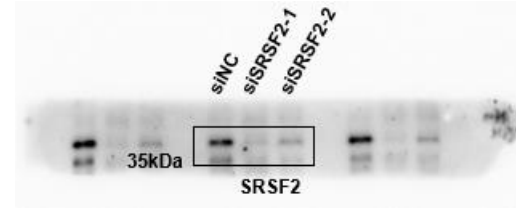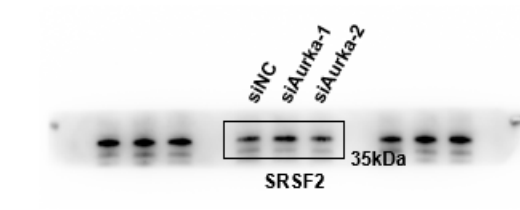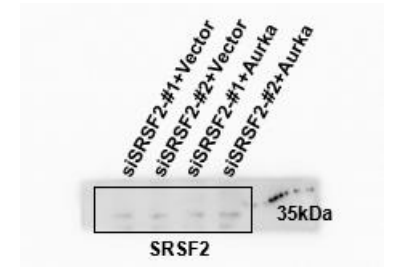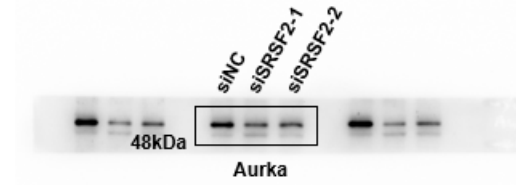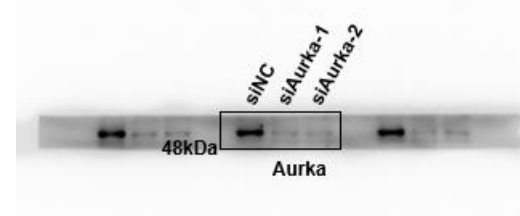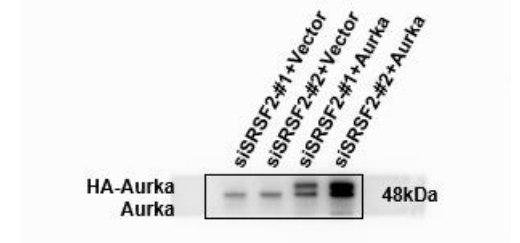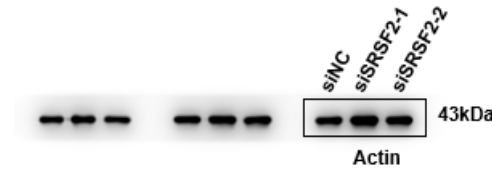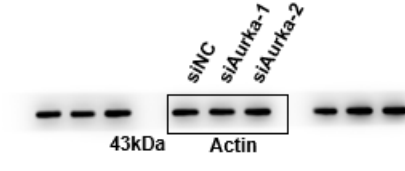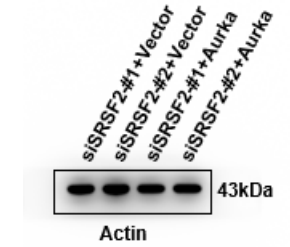

Supplement: Figure 7—source data 4. [file elife-98175-fig7-data4.zip › Figure 7—source data 4/Figure 7H.pdf]

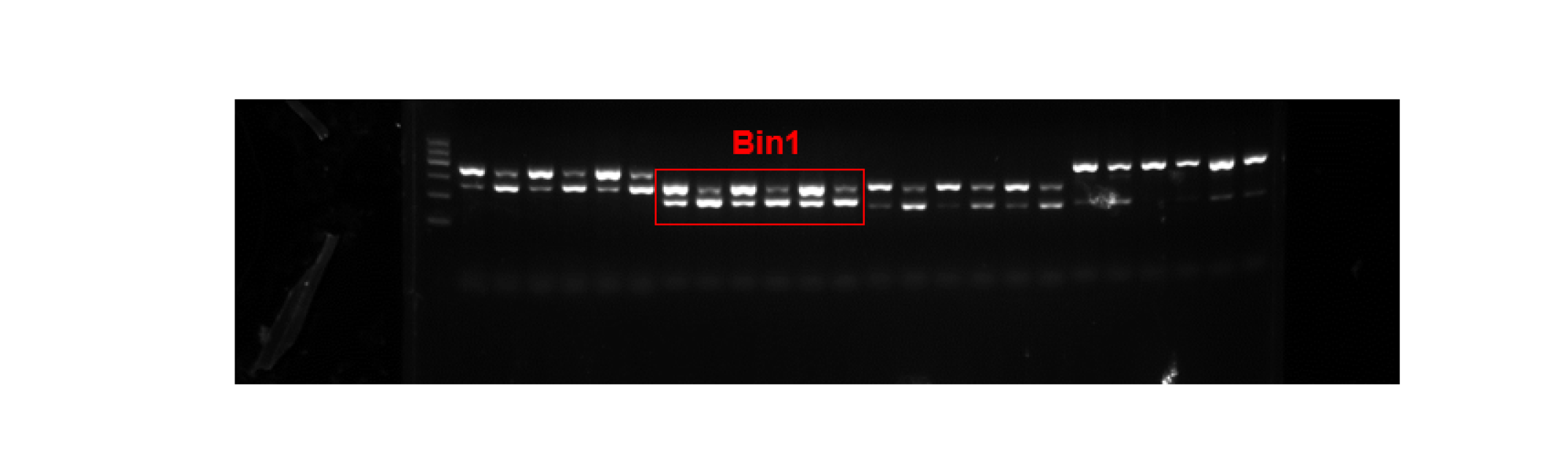

Supplement: Figure 8—source data 1. [file elife-98175-fig8-data1.zip › Figure 8—source data 1/Figure 8B-Bin1.tif]

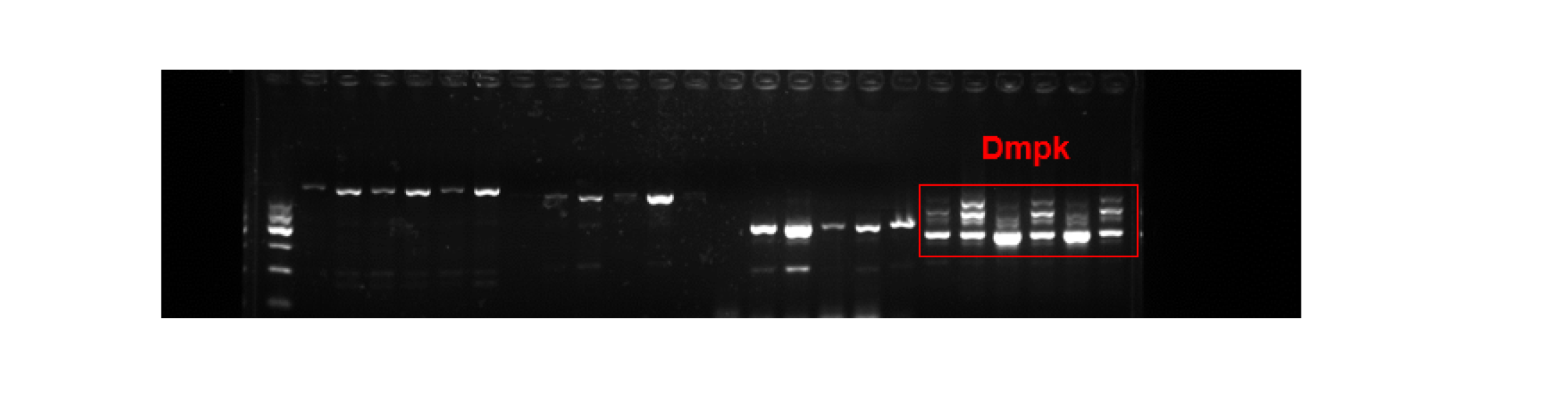

Supplement: Figure 8—source data 1. [file elife-98175-fig8-data1.zip › Figure 8—source data 1/Figure 8B-Dmpk.tif]

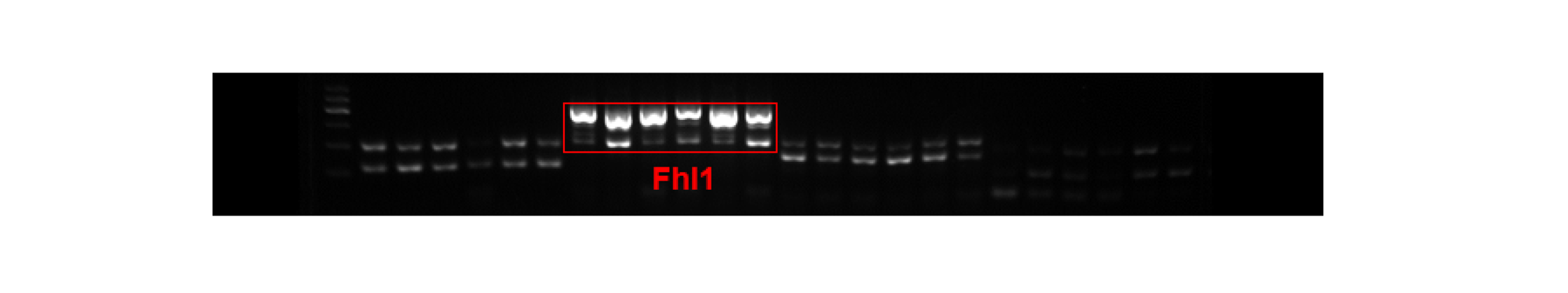

Supplement: Figure 8—source data 1. [file elife-98175-fig8-data1.zip › Figure 8—source data 1/Figure 8B-Fhl1.tif]

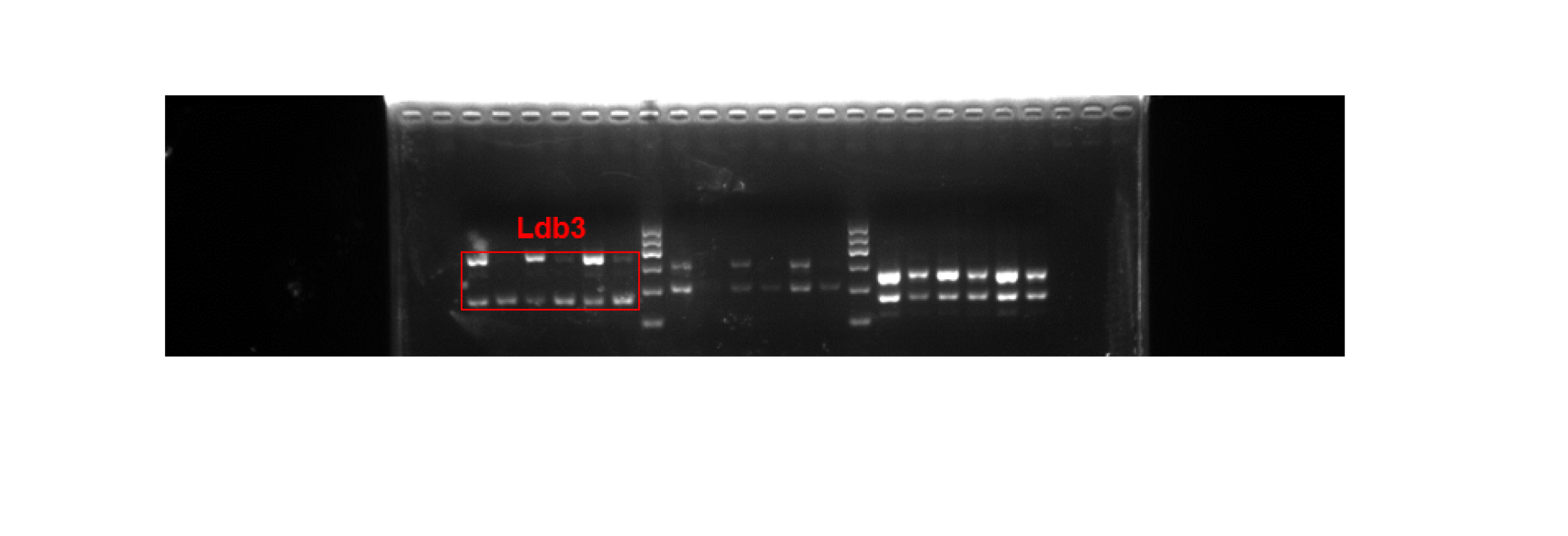

Supplement: Figure 8—source data 1. [file elife-98175-fig8-data1.zip › Figure 8—source data 1/Figure 8B-Ldb3.tif]

Figure 8B

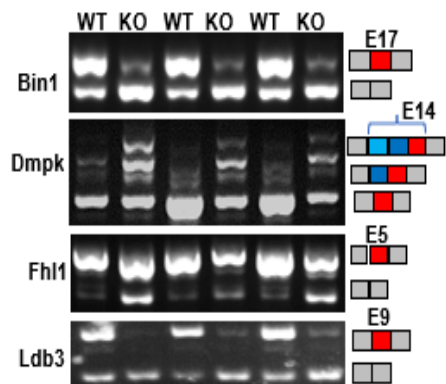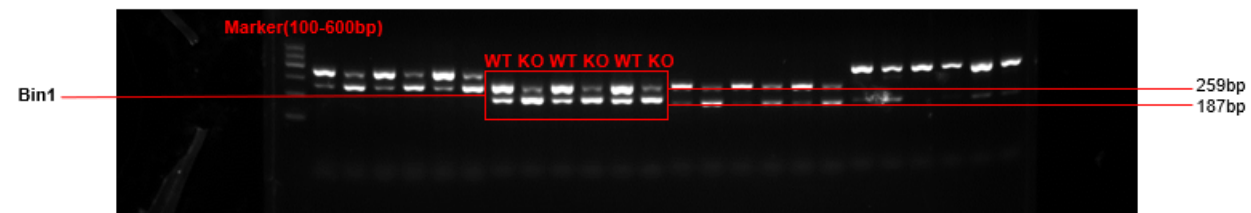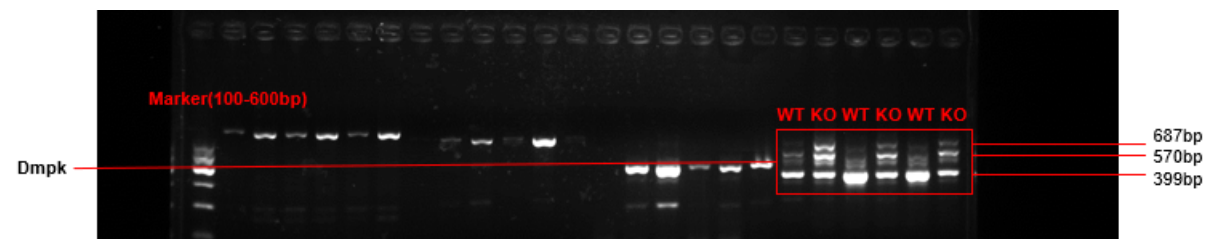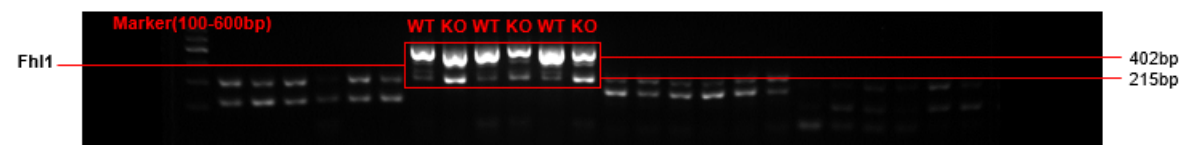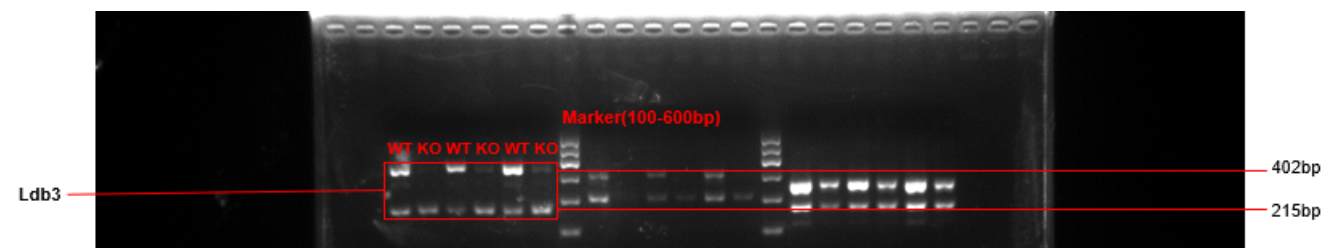

Supplement: Figure 8—source data 2. [file elife-98175-fig8-data2.zip › Figure 8—source data 2/Figure 8B.pdf]

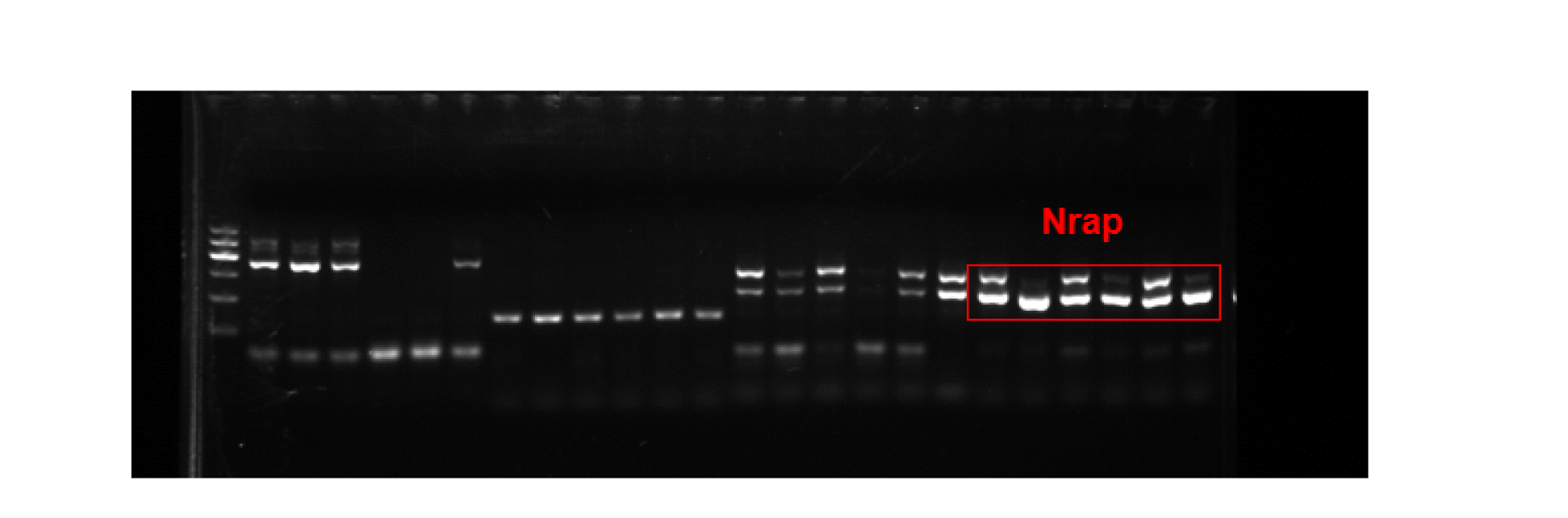

Supplement: Figure 8—source data 3. [file elife-98175-fig8-data3.zip › Figure 8—source data 3/Figure 8C-Nrap.tif]

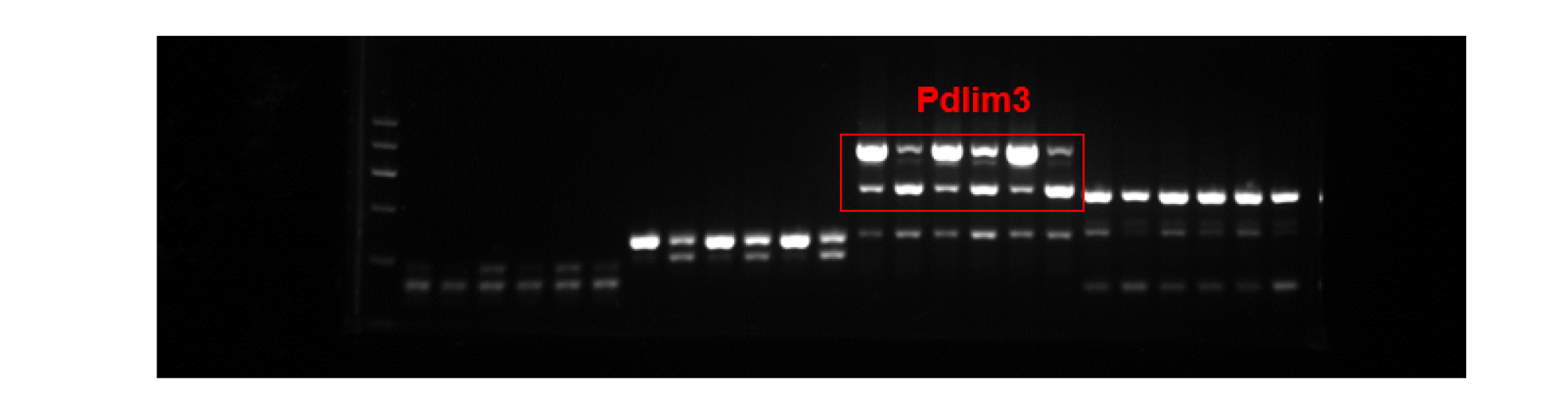

Supplement: Figure 8—source data 3. [file elife-98175-fig8-data3.zip › Figure 8—source data 3/Figure 8C-Pdlim3.tif]

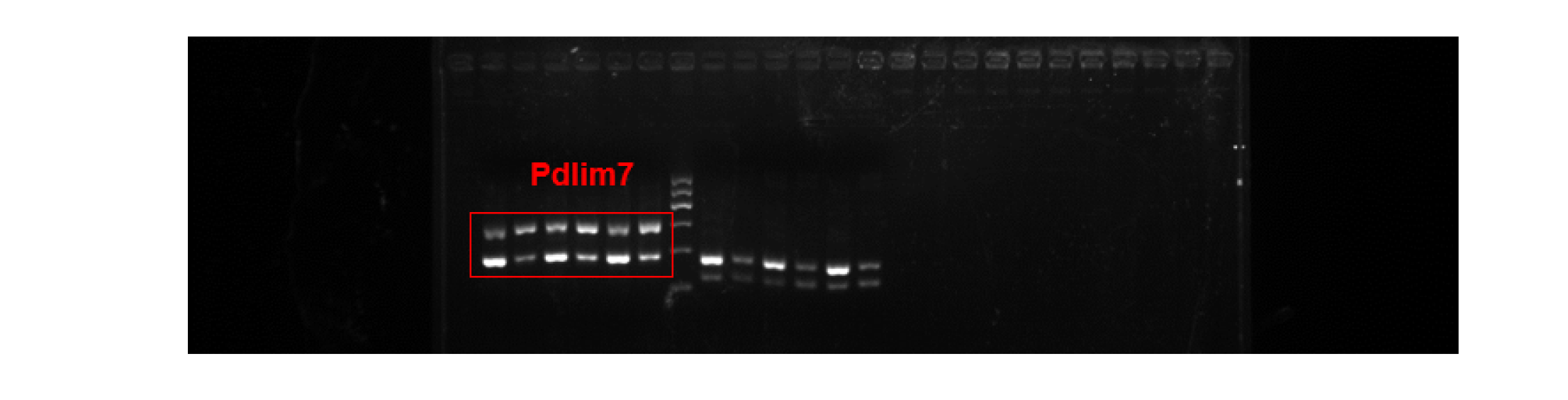

Supplement: Figure 8—source data 3. [file elife-98175-fig8-data3.zip › Figure 8—source data 3/Figure 8C-Pdlim7.tif]

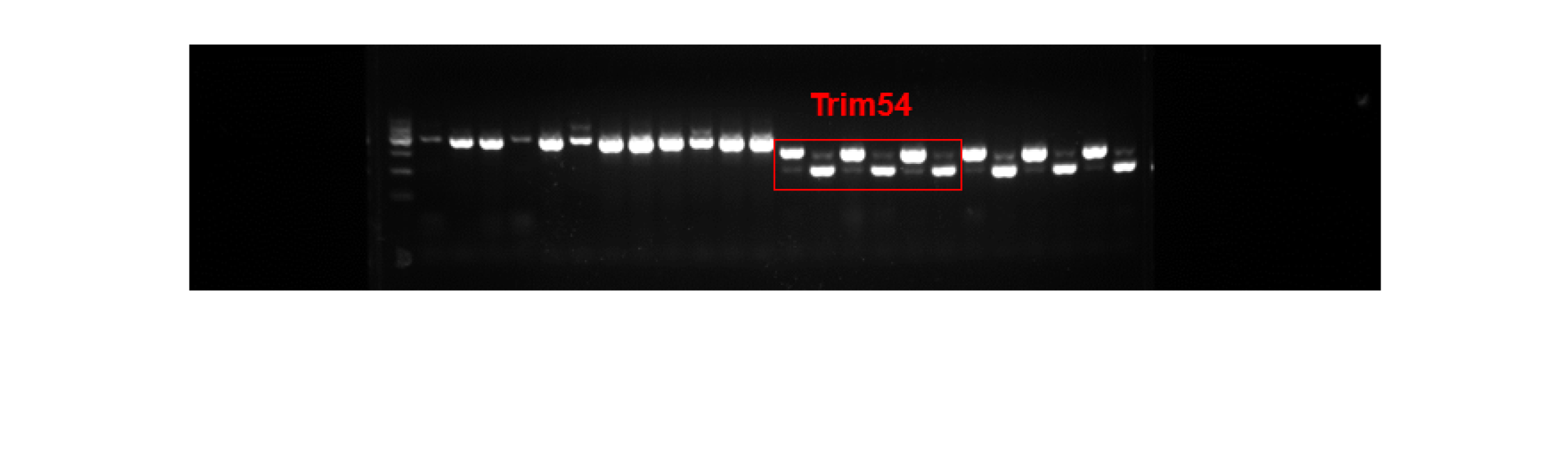

Supplement: Figure 8—source data 3. [file elife-98175-fig8-data3.zip › Figure 8—source data 3/Figure 8C-Trim54.tif]

Figure 8C

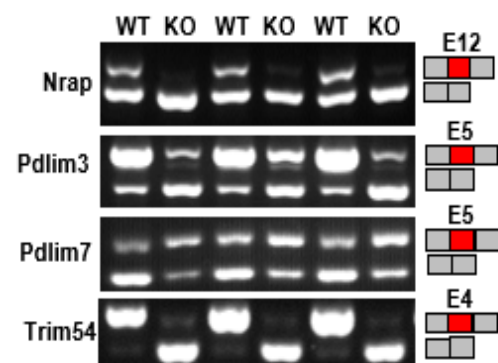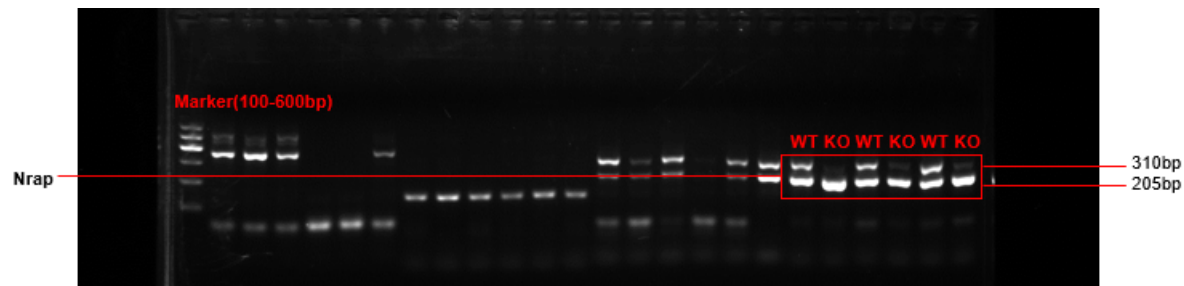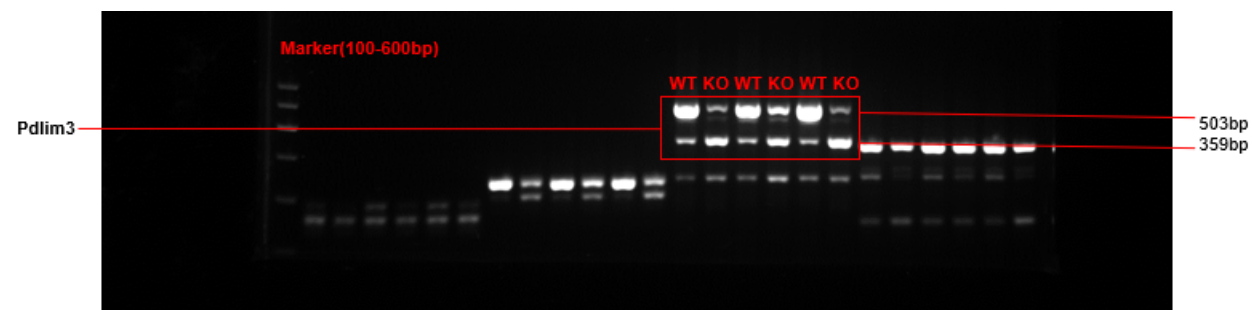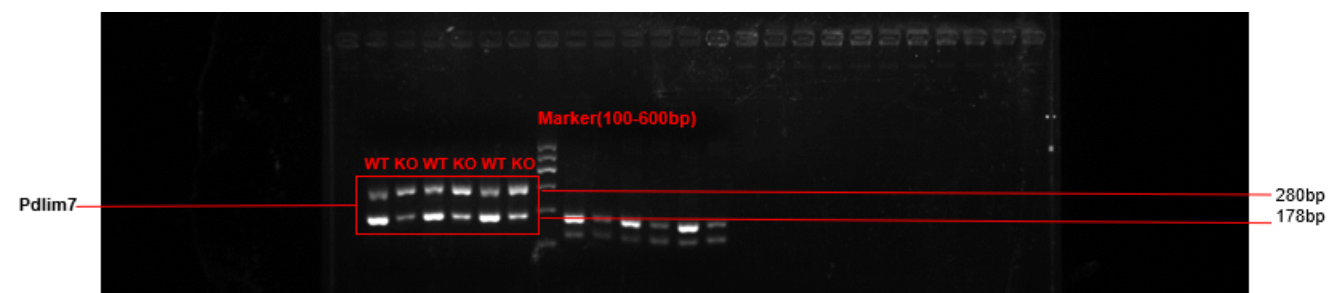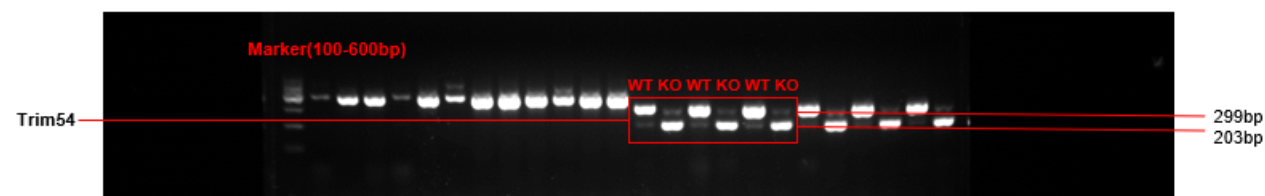

Supplement: Figure 8—source data 4. [file elife-98175-fig8-data4.zip › Figure 8—source data 4/Figure 8C.pdf]

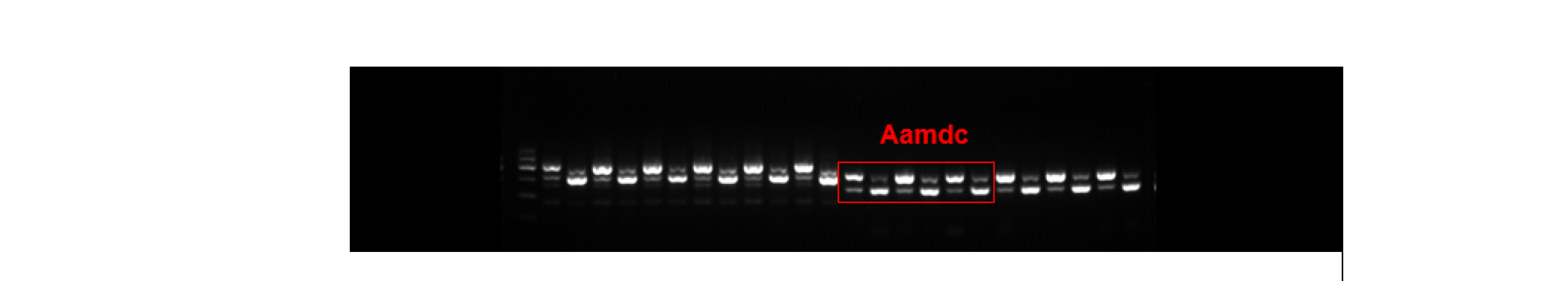

Supplement: Figure 8—source data 5. [file elife-98175-fig8-data5.zip › Figure 8—source data 5/Figure 8D-Aamdc.tif]

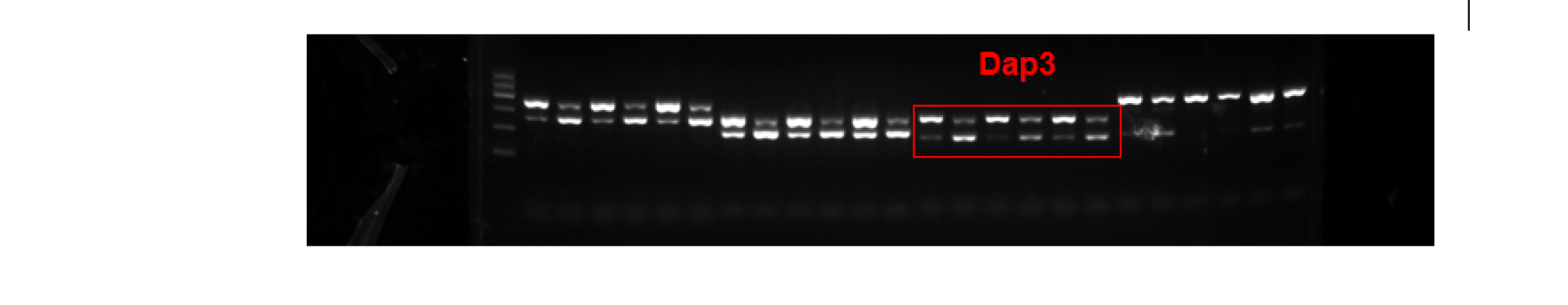

Supplement: Figure 8—source data 5. [file elife-98175-fig8-data5.zip › Figure 8—source data 5/Figure 8D-Dap3.tif]

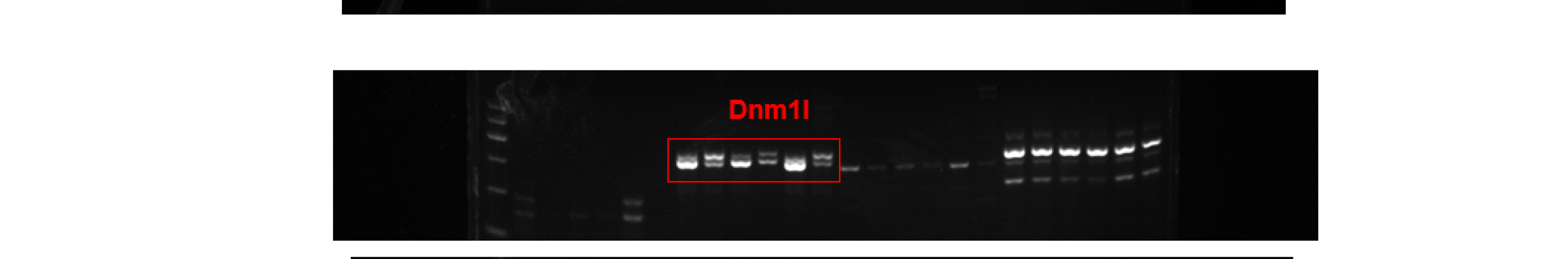

Supplement: Figure 8—source data 5. [file elife-98175-fig8-data5.zip › Figure 8—source data 5/Figure 8D-Dnm1l.tif]

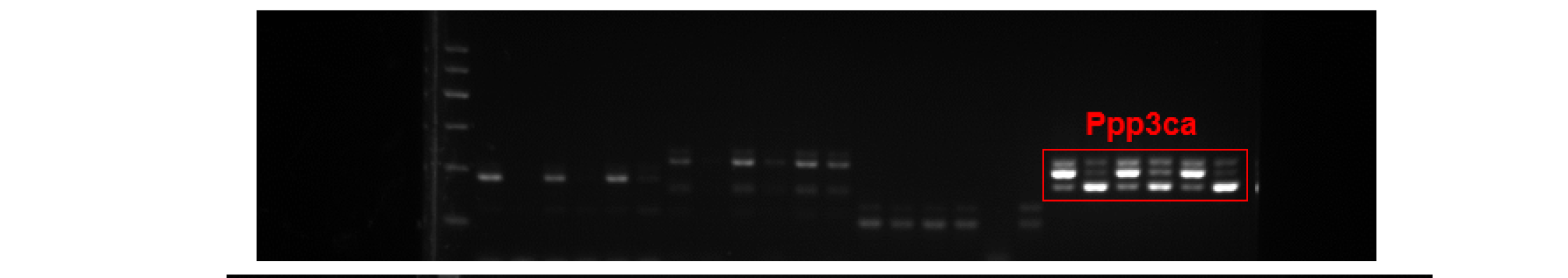

Supplement: Figure 8—source data 5. [file elife-98175-fig8-data5.zip › Figure 8—source data 5/Figure 8D-Ppp3ca.tif]

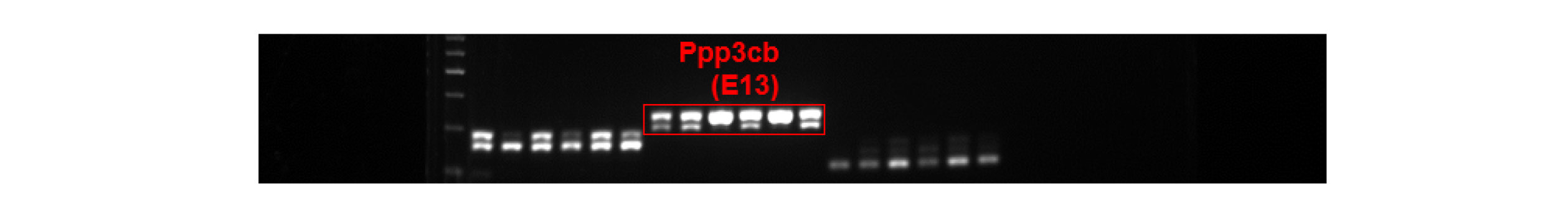

Supplement: Figure 8—source data 5. [file elife-98175-fig8-data5.zip › Figure 8—source data 5/Figure 8D-Ppp3cb (E13).tif]

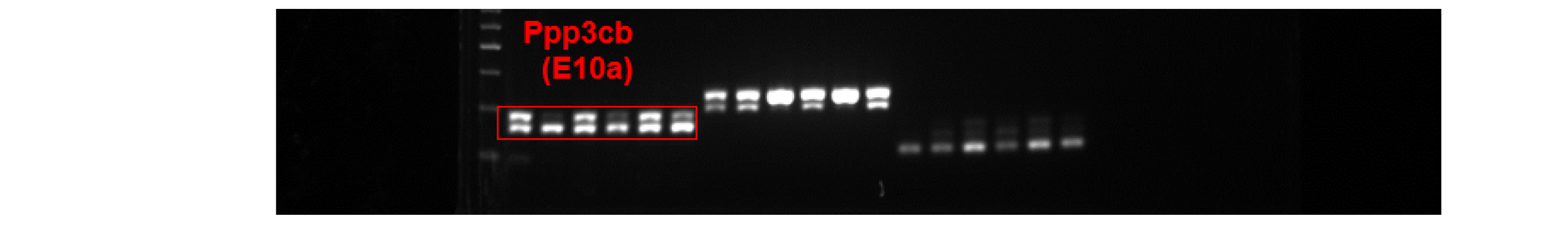

Supplement: Figure 8—source data 5. [file elife-98175-fig8-data5.zip › Figure 8—source data 5/Figure 8D-Ppp3cb(E10a).tif]

Figure 8D

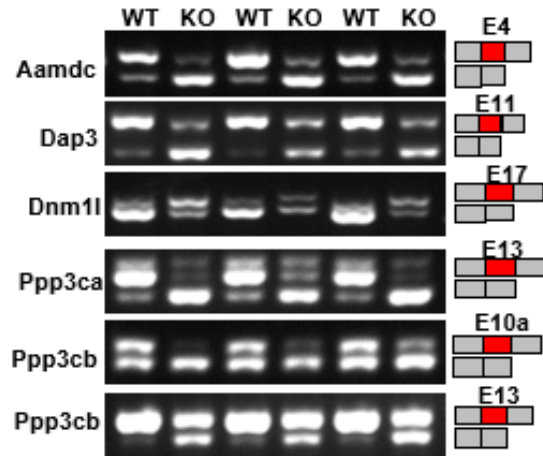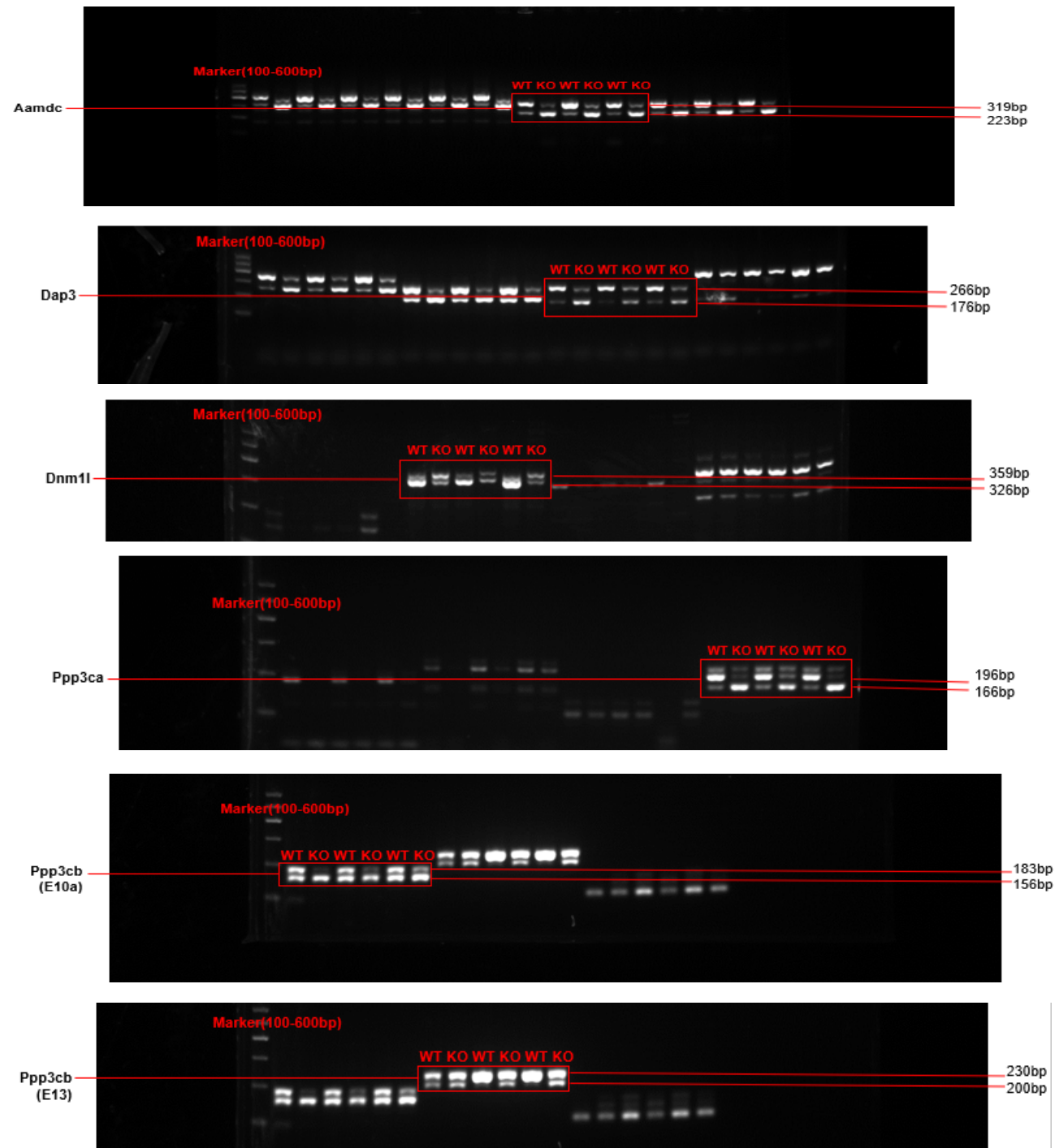

Supplement: Figure 8—source data 6. [file elife-98175-fig8-data6.zip › Figure 8—source data 6/Figure 8D.pdf]

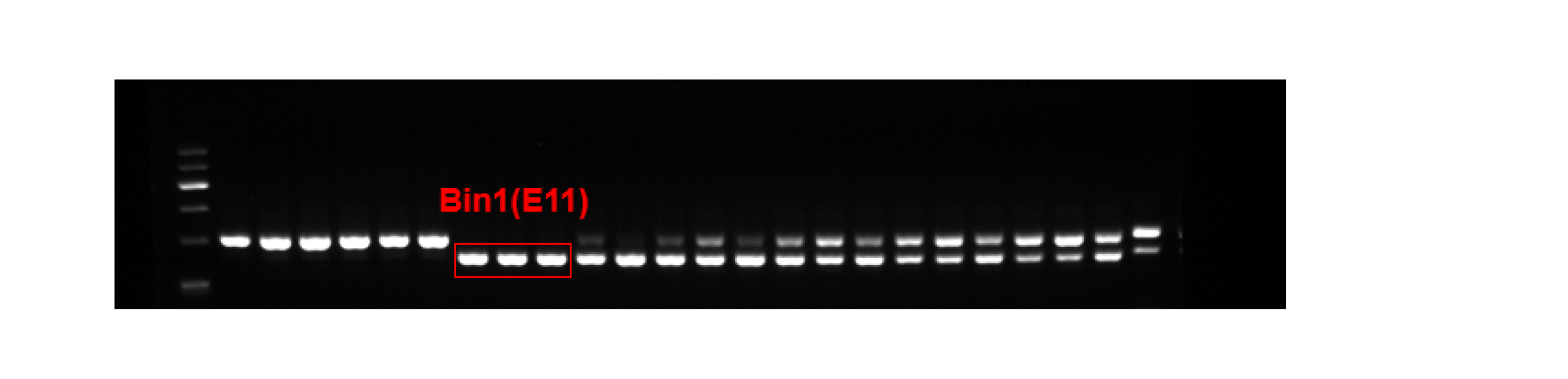

Supplement: Figure 8—source data 7. [file elife-98175-fig8-data7.zip › Figure 8—source data 7/Figure 8E-Bin1(E11).tif]

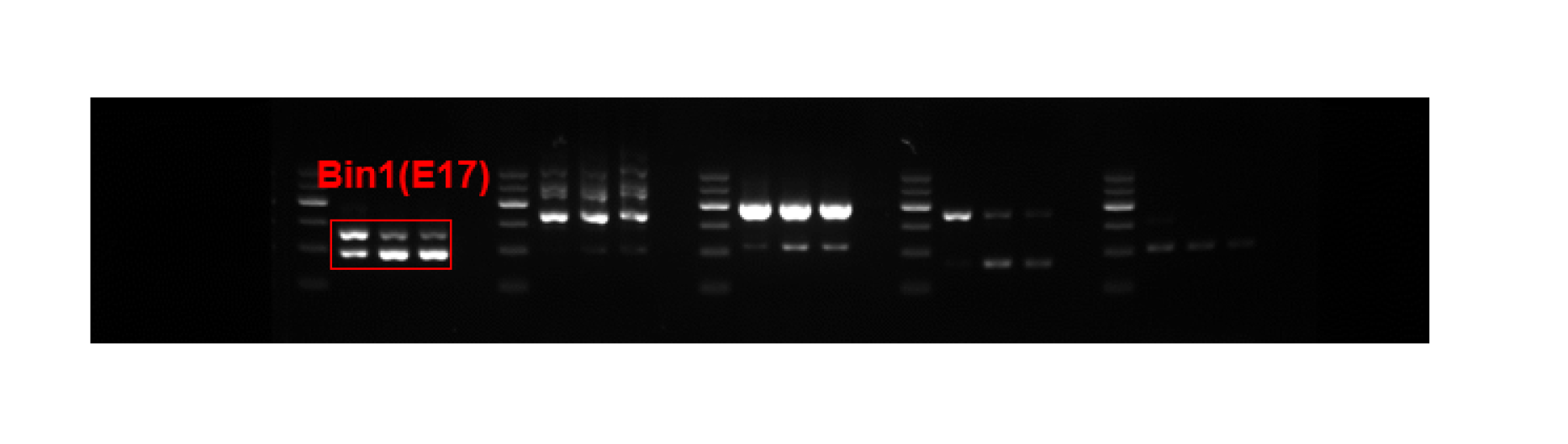

Supplement: Figure 8—source data 7. [file elife-98175-fig8-data7.zip › Figure 8—source data 7/Figure 8E-Bin1(E17).tif]

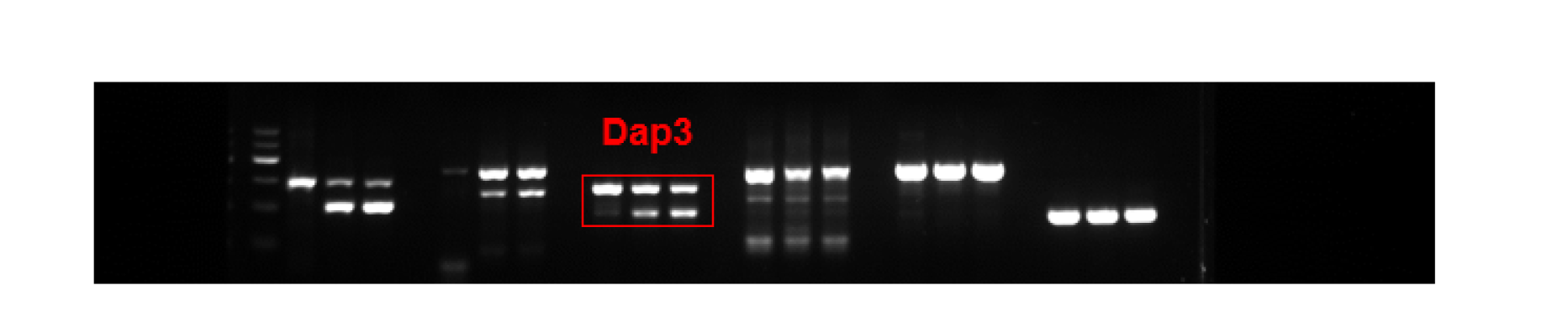

Supplement: Figure 8—source data 7. [file elife-98175-fig8-data7.zip › Figure 8—source data 7/Figure 8E-Dap3.tif]

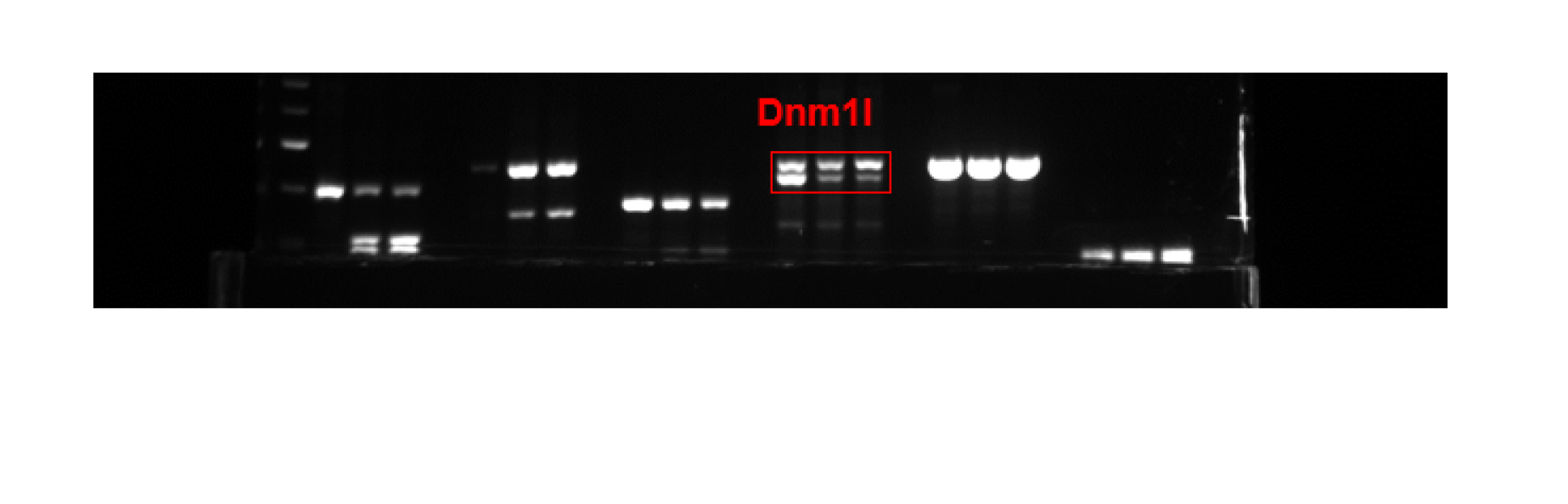

Supplement: Figure 8—source data 7. [file elife-98175-fig8-data7.zip › Figure 8—source data 7/Figure 8E-Dnm1l.tif]

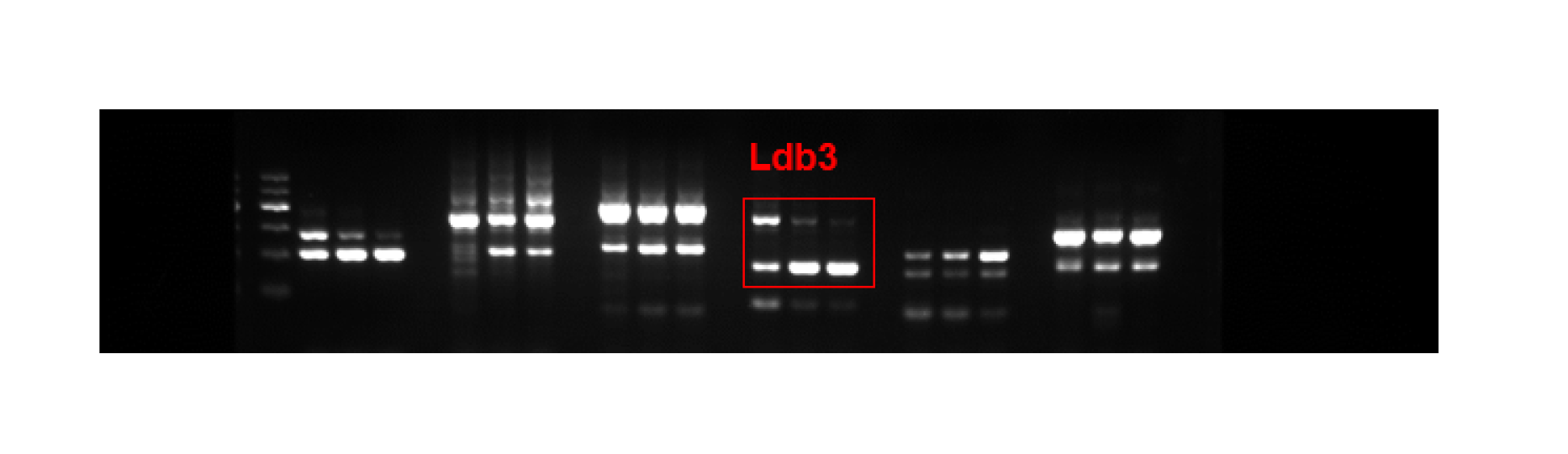

Supplement: Figure 8—source data 7. [file elife-98175-fig8-data7.zip › Figure 8—source data 7/Figure 8E-Ldb3.tif]

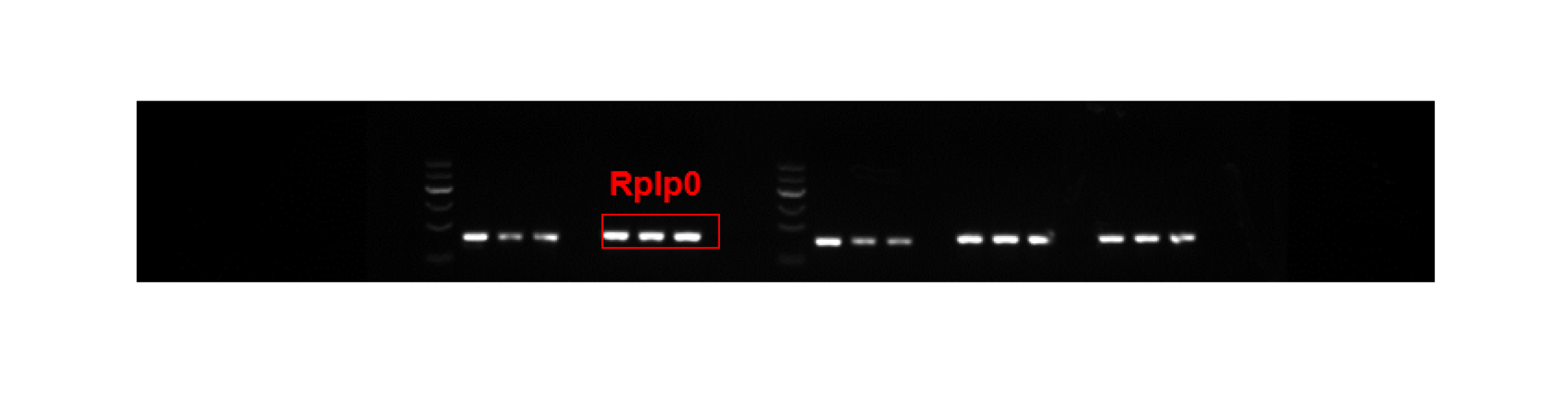

Supplement: Figure 8—source data 7. [file elife-98175-fig8-data7.zip › Figure 8—source data 7/Figure 8E-Rplp0.tif]

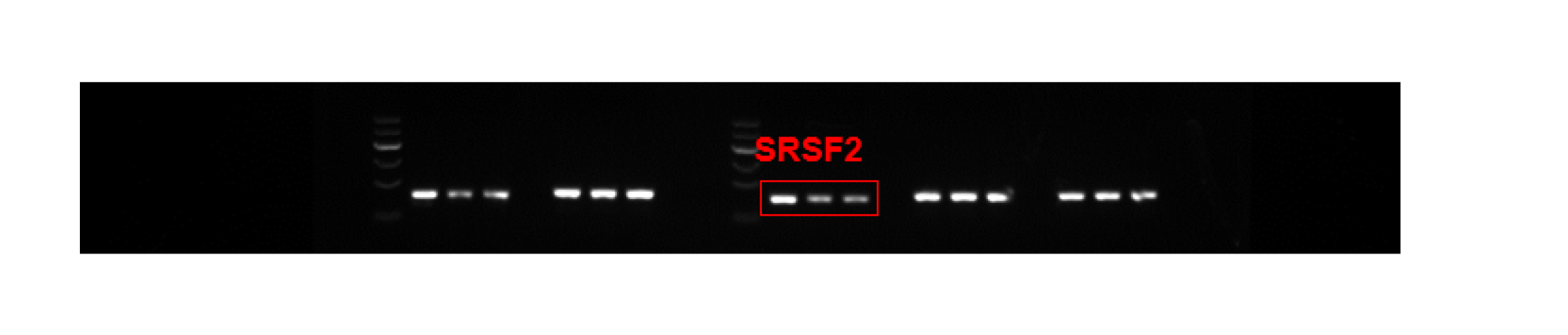

Supplement: Figure 8—source data 7. [file elife-98175-fig8-data7.zip › Figure 8—source data 7/Figure 8E-SRSF2.tif]

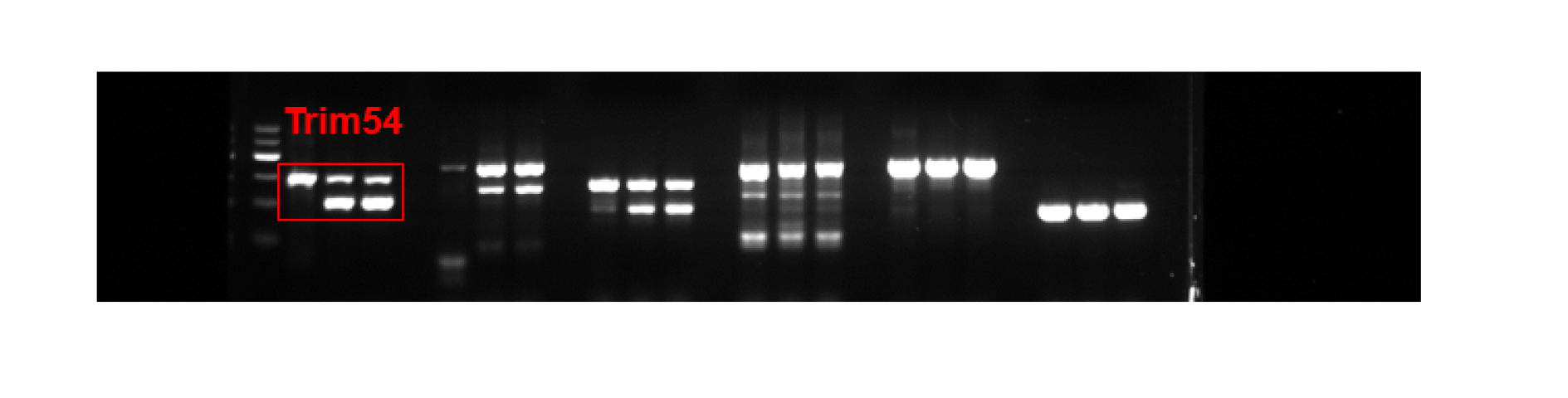

Supplement: Figure 8—source data 7. [file elife-98175-fig8-data7.zip › Figure 8—source data 7/Figure 8E-Trim54.tif]

**Figure 8E**

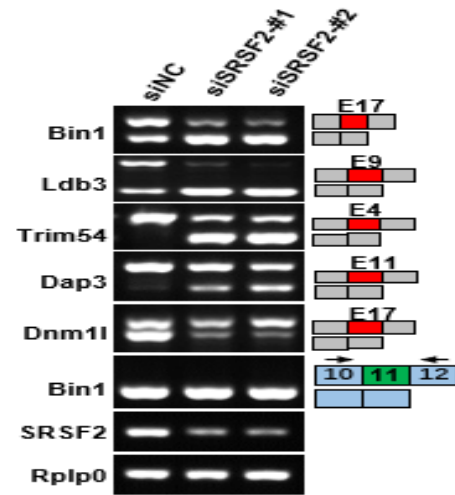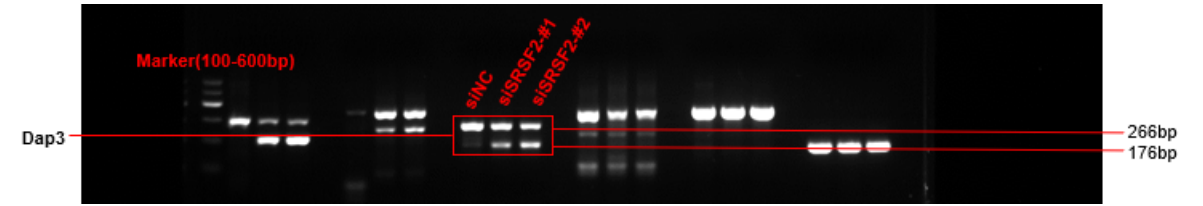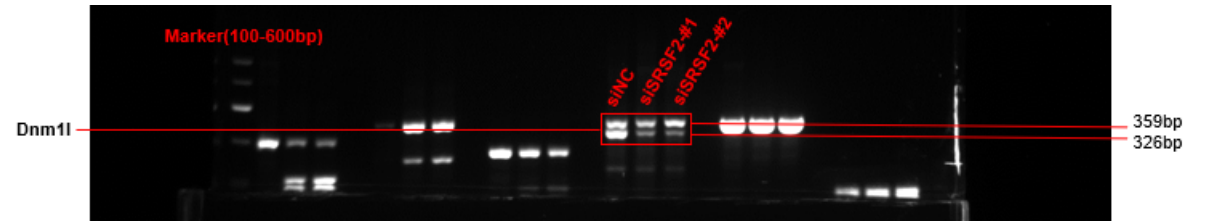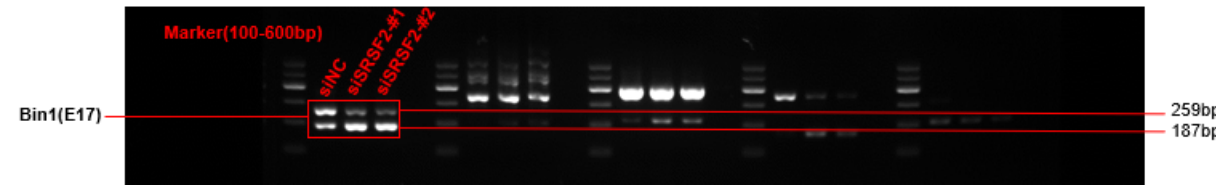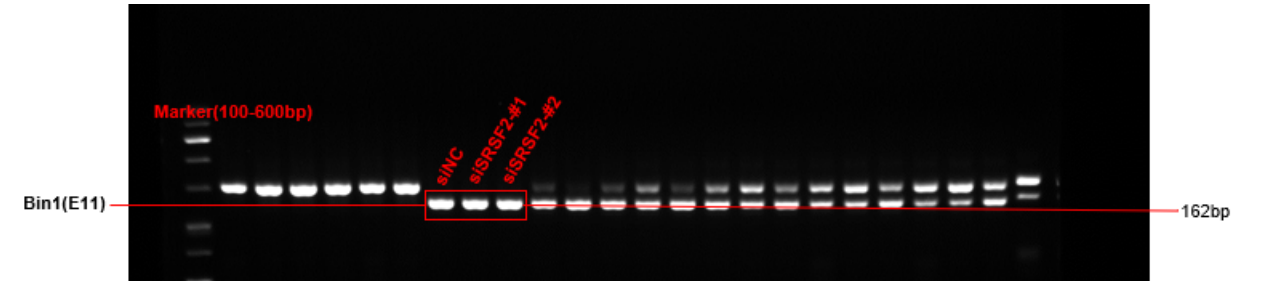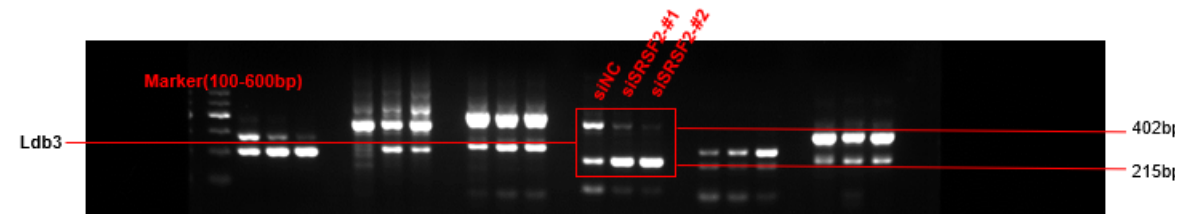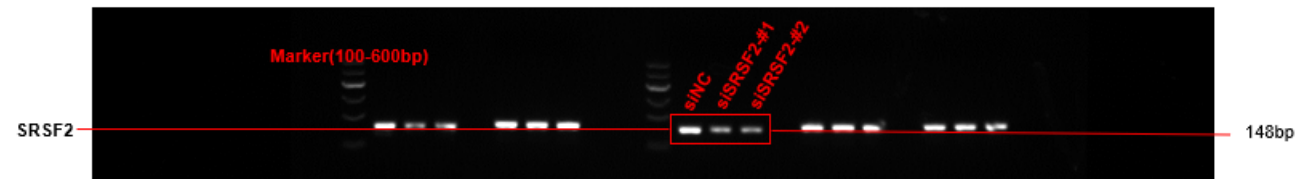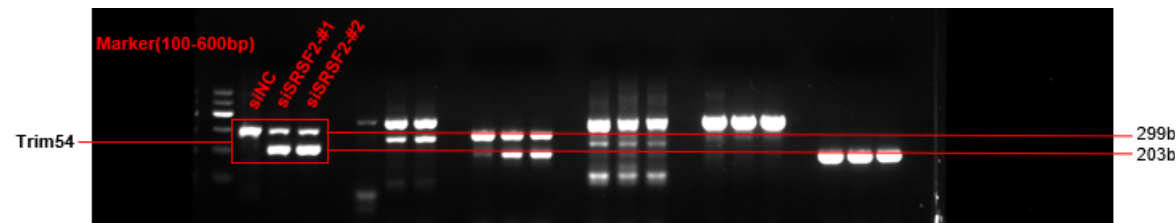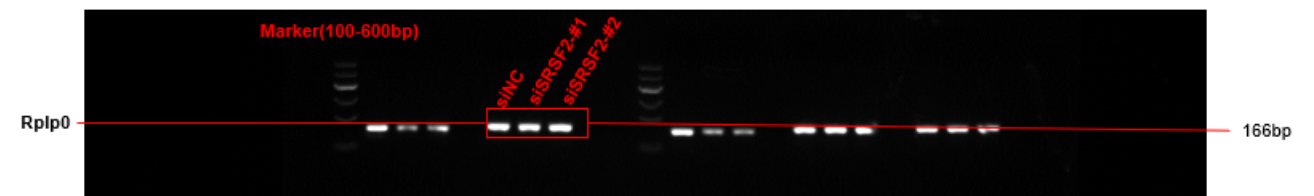

Supplement: Figure 8—source data 8. [file elife-98175-fig8-data8.zip › Figure 8—source data 8/Figure 8E.pdf]

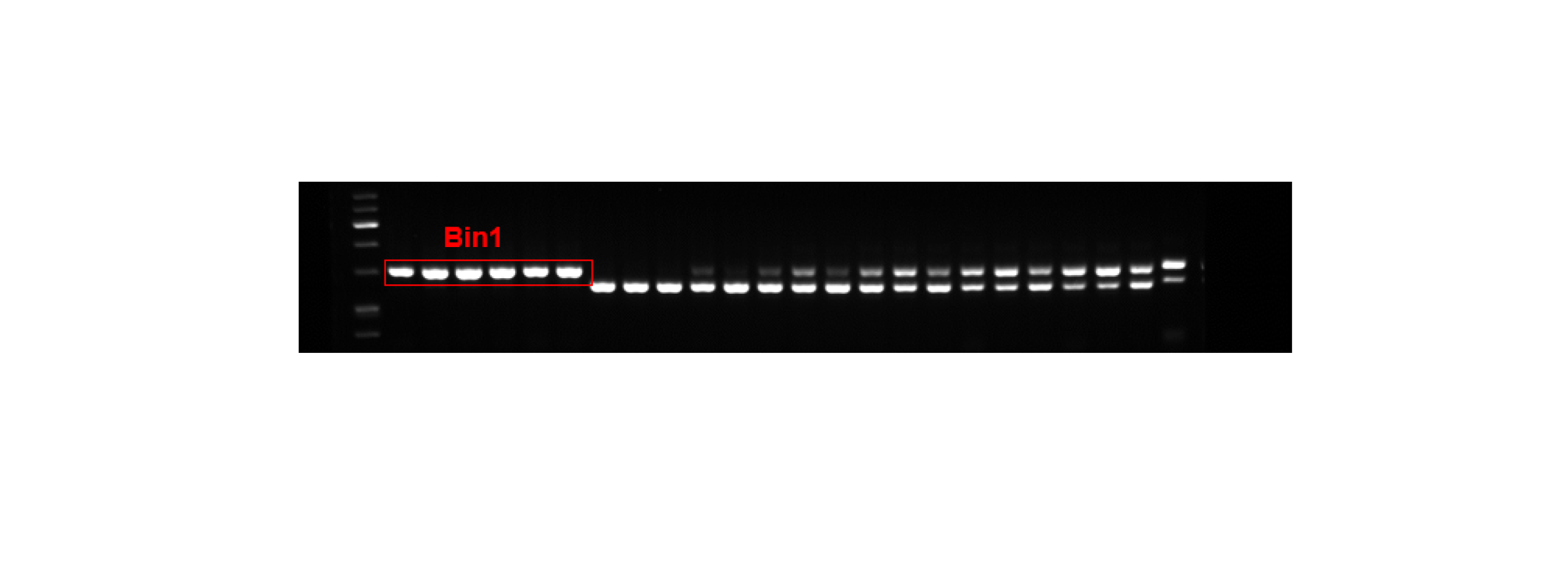

Supplement: Figure 8—source data 9. [file elife-98175-fig8-data9.zip › Figure 8—source data 9/Figure 8G-Bin1.tif]

**Figure 8G**

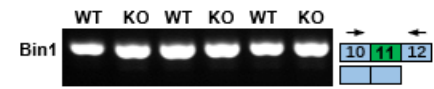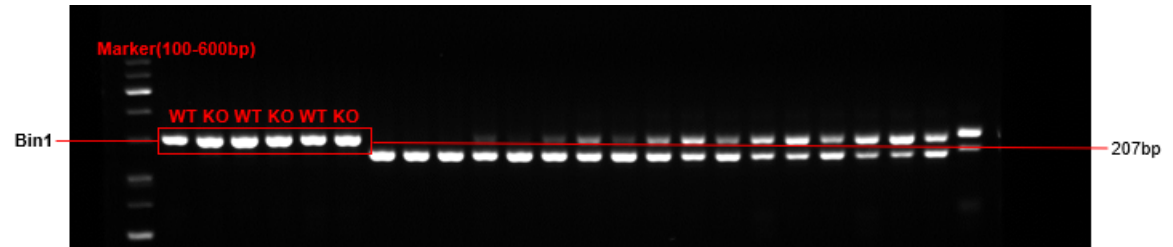

Supplement: Figure 8—source data 10. [file elife-98175-fig8-data10.zip › Figure 8—source data 10/Figure 8G.pdf]

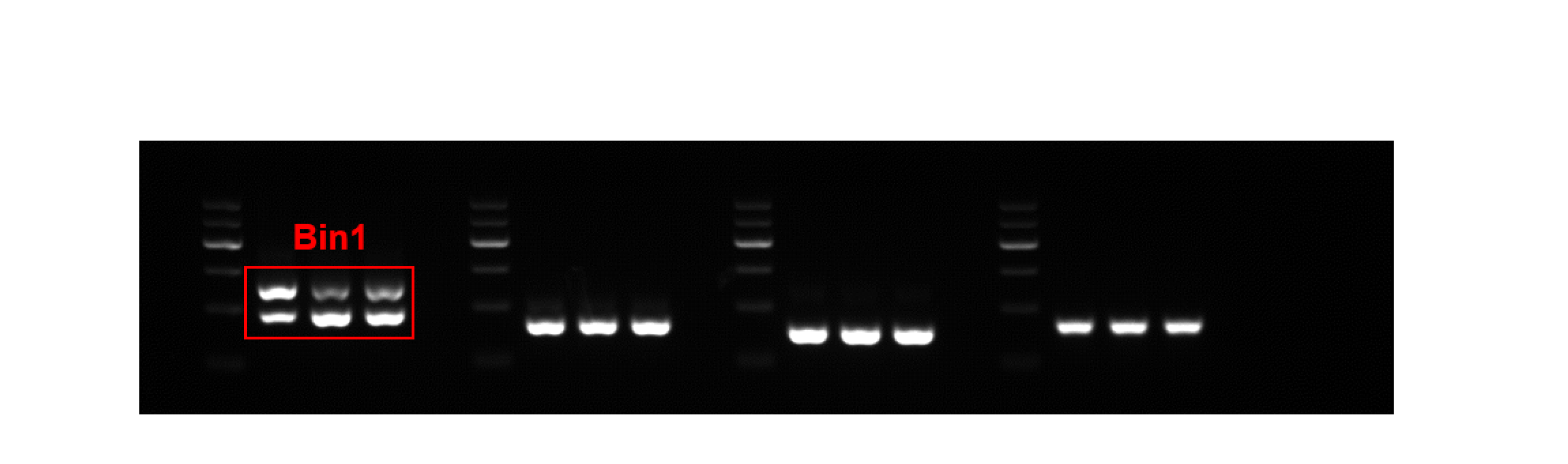

Supplement: Figure 8—source data 11. [file elife-98175-fig8-data11.zip › Figure 8—source data 11/Figure 8H-Bin1.tif]

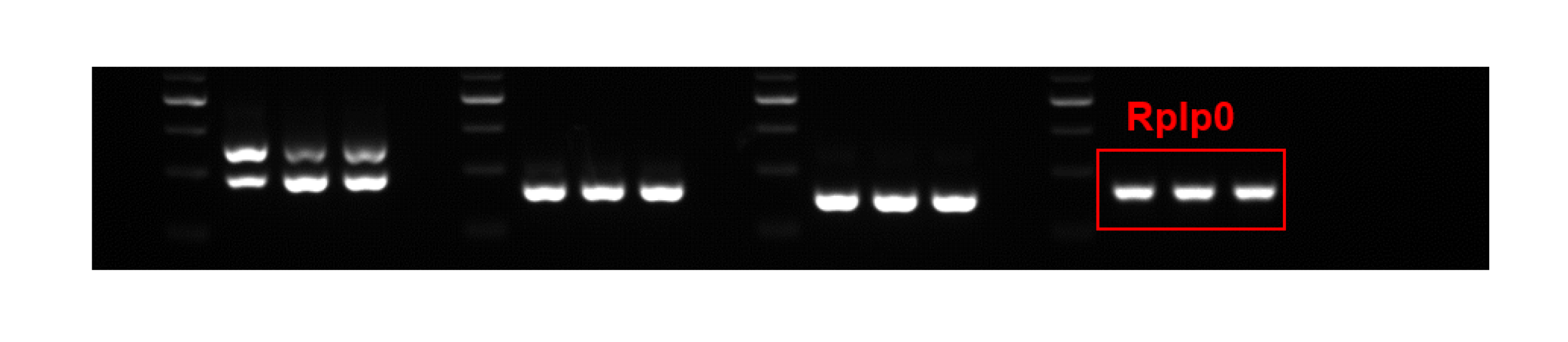

Supplement: Figure 8—source data 11. [file elife-98175-fig8-data11.zip › Figure 8—source data 11/Figure 8H-Rplp0.tif]

Figure 8H

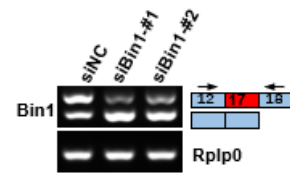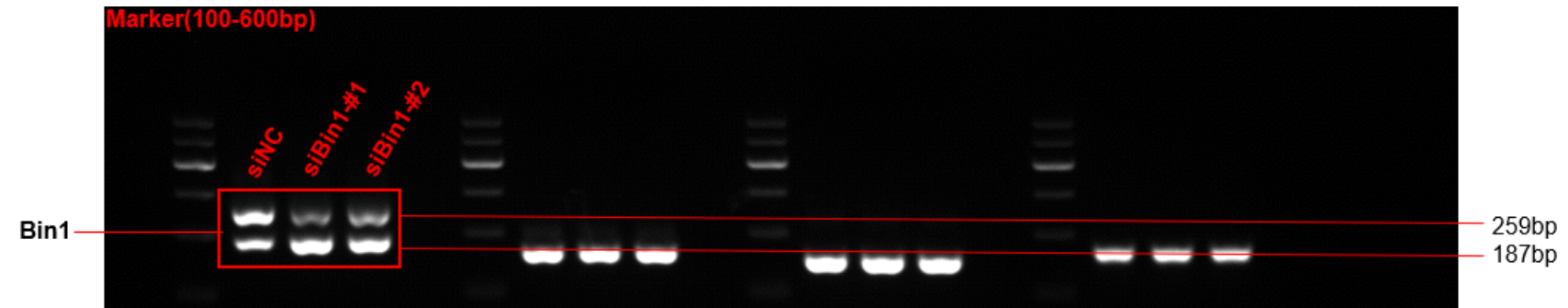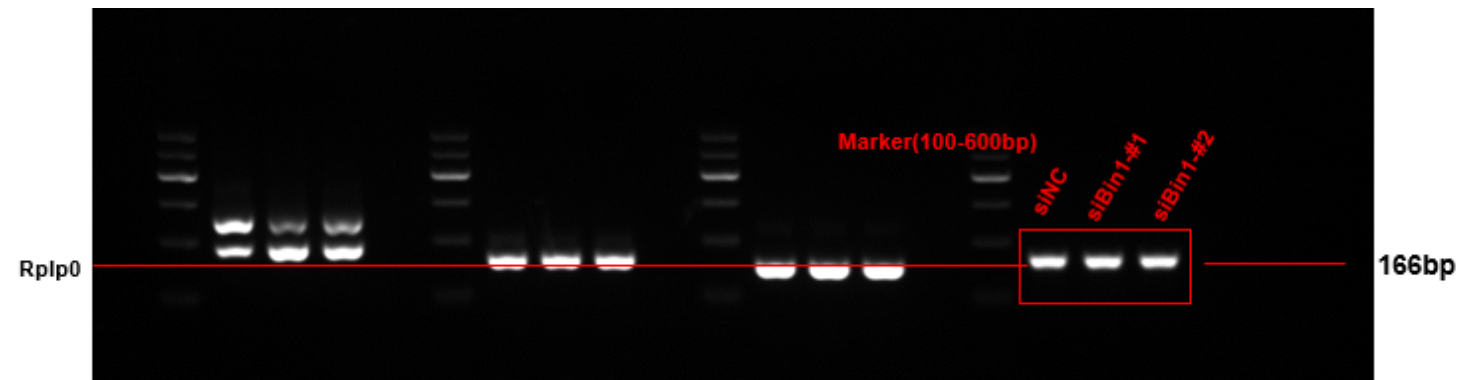

Supplement: Figure 8—source data 12. [file elife-98175-fig8-data12.zip › Figure 8—source data 12/Figure 8H.pdf]
