# Supplementary material for: SRSF2 is a key player in orchestrating the directional migration and differentiation of MyoD progenitors during skeletal muscle development
Source: eLife. 2024 Sep 9;13:RP98175. doi: 10.7554/eLife.98175 (PMC11383525; doi:10.7554/eLife.98175)
Supplement: Supplementary file 1. — (a) Antibodies used in IF, WB, and ChIP analysis. (b) Primer sequences used for RT-PCR. (c) Primer sequence used for qPCR. (d) SiRNA sequence used for RNA interference. (e) Primer sequence used for ChIP-qPCR. [file elife-98175-supp1.docx]

**Supplementary file 1**

**Supplemental file 1a.**

| Myosin heavy chain (MHC) Antibody | DSHB | Cat# mf-20-s | IF |
| --- | --- | --- | --- |
| RFP Antibody | Rockland | Cat# 600-401-379 | IF |
| Phalloidin | YEASEN | Cat# 40762ES75 | IF |
| Laminin-α2 Antibody | ENZO | Cat# ALX-804-  190 | IF |
| Desmin Antibody | Santa Cruz | Cat# sc14026 | IF |
| Myog Antibody | Santa Cruz | Cat#(sc-576) | IF |
| Goat anti-Mouse IgG (H+L) Cross-Adsorbed Secondary antibody, Alexa Fluor 488 | Thermo Fisher Scientific | Cat# A-11001 | IF |
| Goat anti- Rabbit IgG (H+L) Cross-Adsorbed Secondary antibody, Alexa Fluor 546 | Thermo Fisher Scientific | Cat# A-11010 | IF |
| Alexa Fluor® 647-conjugated AffiniPure Donkey Anti-Rat IgG (H+L) | Jackson | Cat# 712-605-150 | IF |
|  |  |  |  |
| SRSF2 | Millipore | Cat# 04-1550 | WB |
| Aurka Antibody | Abcam | Cat# ab13824 | WB |
| CDK1 Antibody | Beyotime | Cat# AF1516 | WB |
| pRB Antibody | Beyotime | Cat# AF1564 | WB |
| p-pRB Antibody | Beyotime | Cat# AF1135 | WB |
| p27 Antibody | Cell Signaling Technology | Cat# 3698 | WB |
| AKT Antibody | Cell Signaling Technology | Cat# 4691 | WB |
| p-AKT Antibody | Cell Signaling Technology | Cat# 4060 | WB |
| p62 Antibody | Sigma-Aldrich | Cat# P0067 | WB |
| β-Actin Antibody | Santa Cruz | Cat# sc47778 | WB |
| Anti-HA tag | Abcam | Cat# ab9110 | ChIP |

**Supplemental file 1b.**

| Gene | Forward (5’–3’) | Reverse (5’–3’) |
| --- | --- | --- |
| *Bin1*-E17 | CCCTGAGAAAGGGAACAAGA | ATTCACAGTTGCGGAGAAGG |
| *Bin1*-E11 | TCAATGATGTCCTGGTCAGC | GCTCATGGTTCACTCTGATC |
| *Dmpk* | AACTACAGGAGGCCGAGGTC | TATAGGCCACCAACCCAGTG |
| *Fhl1* | GAACGTGGAGTACAAGGGCA | TCGTGCCAGGATTGTCCTTC |
| *Ldb3* | GCAAGACCCTGATGAAGAGG | GATGCTGGCAGTGGTTACG |
| *Nrap* | ACCGGCAGGACTTCCATAAG | CTGCCAACTTTGAGAGCGTG |
| *Pdlim3* | GAAAAGCACACCCCTTCAAA | CCCGTCATTCACCAGATCCT |
| *Pdlim7* | CCTGTTCAGAGCAAACCACA | TGAACTCTGTGCCCGTGA |
| *Trim54* | CCTGCTCTCTCTGCAAGGTT | CTGCAACTTCTCCTCCTGCT |
| *Aamdc* | GGACAAATGAAAGTGCAAGGCT | AGACACCTCCTACCCTGACC |
| *Dap3* | ATTGCCCCAGAGGAACTCTC | TTCTCATGTTGAAGCCAGTTG |
| *Dnm1l* | CTGCTTCTGCTGAGGCTGAT | TGCCTTTGGGACACTGTCTT |
| *Ppp3ca* | CTGACACTGAAGGGCCTGAC | GAGGTGGCATCCTCTCGTTA |
| *Ppp3cb* ex10a | TTGGTTGCCCAATTTTATGG | CGGGCTGCAGCTGAACCTA |
| *Ppp3cb* ex13 | ACAGGGATGTTGCCTAGTGG | TCAGTGGTATGTGCGGTGTT |

**Supplemental file 1c.**

| Gene | Forward (5’–3’) | Reverse (5’–3’) |
| --- | --- | --- |
| *Srsf2* | ACCTCCCTCAAGGTGGACAA | TTGTCGTGGAACCGGACG |
| *Aurka* | ATGACGCCACCCGAGTTTAT | GCTGTTCTCTGCTCGTCAAA |
| *Fbxo5* | CAGCATCCCAACAAAACCTT | GCTCAGCCAGGATGTCTAGG |
| *Nuf2* | TGGATTGCTTGCCTTCCTGT | TCTGGTCCTCCAAGTTCAAGC |
| *Poc1a* | CTGCTACCACCGTTGCCTTT | TGGTGGGACAGGCAAATCCA |
| *Ranbp1* | AGATGAGTCCAACCACGACC | TTGACATCTCCAGTGCCTCG |
| *Spdl1* | TAGACTCCCACCTCACAGGG | ATGTTGGCCGGACTATCCAC |
| *Scn4b* | GCTTCCCATGTACCTGTCGT | ATCCTGGATGTTTCGCTGTT |
| *Myh1* | TTCATCAGGATCCACTTTGG | CGATTAGATCCGGCTTCTTG |
| *Myh2* | TGCTGACAAAGCTGCCTATC | ACAGTCTGGCCTTTGGTGAC |
| *Synm* | CTGGAGGATGAGAAGGAAGC | TGGCTACCTCCAAGCTGAGT |
| *Tmod4* | CCTGAGACAACGTGACCAGA | CAGTGTAGGGCACCAAGTCA |
| *Rplp0* | TAAAGACTGGAGACAAGGTGGGAG | AGAAAGCGAGAGTGCAGGGC |

**Supplemental file 1d.**

|  | Sense | Antisense |
| --- | --- | --- |
| siSrsf2 -#1 | CGAAGAUCCAAGUCCAAGUTT | ACUUGGACUUGGAUCUUCGTT |
| siSrsf2-#2 | UCCAGAUCAACCUCCAAGUTT | ACUUGGAGGUUGAUCUGGATT |
| siAurka-#1 | GAGCAGGUCAGAAGCCGGCACCAAA | UUUGGUGCCGGCUUCUGACCUGCUC |
| siAurka-#2 | CCAGAAACUUGGAGCAGGUCAGAAG | CUUCUGACCUGCUCCAAGUUUCUGG |
| siBin1-#1 | CAGCCAGUGAAGCAACCUCTT | GAGGUUGCUUCACUGGCUGTT |
| siBin1-#2 | CAGUGAAGCAACCUCCAGCTT | GCUGGAGGUUGCUUCACUGTT |
| siNC | UUCUCCGAACGUGUCACGUTT | ACGUGACACGUUCGGAGAATT |

**Supplementary file 1e.**

| Gene | Forward (5’–3’) | Reverse (5’–3’) |
| --- | --- | --- |
| Aurka-ChIP | AAGGGACATGGCTGTTGAGG | ACAGAATGCAAACCCACTGAGA |
